# Identification of Hammerhead-variant ribozyme sequences in SARS-CoV-2

**DOI:** 10.1093/nar/gkae037

**Published:** 2024-01-31

**Authors:** Getong Liu, Hengyi Jiang, Dongrong Chen, Alastair I H Murchie

**Affiliations:** Shanghai Pudong Hospital, Fudan University Pudong Medical Center and Institutes of Biomedical Sciences, Shanghai Medical College, Key Laboratory of Medical Epigenetics and Metabolism, Fudan University, Shanghai 200032, China; Key Laboratory of Metabolism and Molecular Medicine, Ministry of Education, School of Basic Medical Sciences, Fudan University, Shanghai 200032, China; Shanghai Pudong Hospital, Fudan University Pudong Medical Center and Institutes of Biomedical Sciences, Shanghai Medical College, Key Laboratory of Medical Epigenetics and Metabolism, Fudan University, Shanghai 200032, China; Key Laboratory of Metabolism and Molecular Medicine, Ministry of Education, School of Basic Medical Sciences, Fudan University, Shanghai 200032, China; Shanghai Pudong Hospital, Fudan University Pudong Medical Center and Institutes of Biomedical Sciences, Shanghai Medical College, Key Laboratory of Medical Epigenetics and Metabolism, Fudan University, Shanghai 200032, China; Key Laboratory of Metabolism and Molecular Medicine, Ministry of Education, School of Basic Medical Sciences, Fudan University, Shanghai 200032, China; Shanghai Pudong Hospital, Fudan University Pudong Medical Center and Institutes of Biomedical Sciences, Shanghai Medical College, Key Laboratory of Medical Epigenetics and Metabolism, Fudan University, Shanghai 200032, China; Key Laboratory of Metabolism and Molecular Medicine, Ministry of Education, School of Basic Medical Sciences, Fudan University, Shanghai 200032, China

## Abstract

The SARS-CoV-2 RNA virus and variants, responsible for the COVID-19 pandemic has become endemic, raised a need for further understanding of the viral genome and biology. Despite vast research on SARS-CoV-2, no ribozymes have been found in the virus genome. Here we report the identification of 39 Hammerhead-variant ribozyme sequences (CoV-HHRz) in SARS-CoV-2. These sequences are highly conserved within SARS-CoV-2 variants but show large diversity among other coronaviruses. *In vitro* CoV-HHRz sequences possess the characteristics of typical ribozymes; cleavage is pH and ion dependent, although their activity is relatively low and Mn^2+^ is required for cleavage. The cleavage sites of four CoV-HHRz coincide with the breakpoint of expressed subgenomic RNA (sgRNAs) in SARS-CoV-2 transcriptome data suggesting *in vivo* activity. The CoV-HHRz are involved in processing sgRNAs for ORF7b, ORF 10 and ORF1ab nsp13 which are essential for viral packaging and life cycle.

## Graphical Abstract

**Graphical Abstract:**
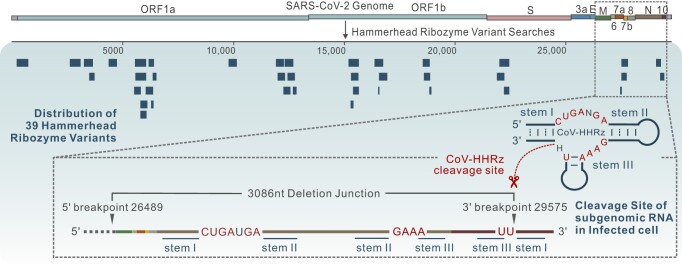

## Introduction

The severe acute respiratory syndrome coronavirus (SARS-CoV-2) has been responsible for the worldwide COVID-19 pandemic since the end of 2019, resulting in hundreds of millions of infections, more than six million deaths, and had a devastating impact on human health and society. SARS-CoV-2 is a positive single strand RNA virus and its genome contains 29 903 nucleotides, which became publicly available soon after its emergence (1–3). There are 4 types of coronavirus (alpha Beta Gamma, Delta) (4). SARS-CoV-2, is a beta coronavirus, a member of the Coronaviridae family that includes coronaviruses that infect birds, pangolins and mammals including pigs and bats (5). Since 2002, coronaviruses have caused successive outbreaks of severe acute respiratory syndrome, including SARS-CoV-1 and middle east respiratory syndrome (MERS-CoV) (6). From the beginning of the COVID-19 pandemic, SARS-CoV-2 variant viruses have emerged including alpha, beta, gamma, delta, lambda and omicron (7). The SARS-CoV-2 genomic RNA encodes essential proteins for the viral life cycle and viral infection (8). The RNA genome includes 2 open reading frames (ORFs), ORF1a and ORF1b that encode 16 nonstructural proteins (nsp1 to nsp16) including an RNA-dependent RNA polymerase (nsp12), RNA helicase (nsp13) and exoribonuclease (nsp14). The RNA genome also encodes spike and viral coat proteins (9). In common with other positive-strand RNA viruses, coronavirus genome replication is a continuous synthesis process. The positive sense viral RNA genome serves as a template for replication and translation (10,11). The resulting negative-sense RNA, encompassing both the full-length genomic RNA (-gRNA) and subgenomic RNAs (-sgRNAs) are transcribed in a discontinuous manner, and are utilized as templates for the synthesis of positive genomic RNAs (gRNAs) and subgenomic RNAs (sgRNAs). The small sgRNAs encode structural proteins, nucleocapsid, and accessory proteins (3a, 6, 7a, 7b, 8 and 10). High-throughput sequencing analysis has yielded high-resolution maps of the SARS-CoV-2 transcriptome and demonstrate that a majority of the sgRNAs are generated by a canonical transcription regulatory sequence (TRS)-mediated template-switching mechanism through discontinuous transcription (8,12–19). In addition, a minority of sgRNAs are generated by non-canonical TRS-dependent mechanisms. Furthermore some sgRNAs contain small sequence deletions through a TRS-independent mechanism that remains unknown (15,20).

The hammerhead ribozymes are nucleolytic ribozymes that catalyse the intramolecular self-cleavage of RNA. They were first identified as components of plant viroids and satellite RNAs (21,22), but are not limited to viral RNAs, and are recognised, as being amongst the most widely distributed nucleolytic ribozyme classes with examples across all three kingdoms of life (23–30). The wide dispersion of the hammerhead ribozymes and their abundance in *in vitro* selection experiments for RNA self-cleavage suggest a pattern of recurrent independent evolution of the hammerhead ribozyme (31).

The hammerhead ribozyme is comprised of 3 helical stems (stems I/II/III) and a catalytic core of ∼11 conserved nucleotides that are essential for catalysis. There are three types of hammerhead ribozyme (type I, II and III). Circular permutation of the three helices about the core nucleotides also contributes to the diversity of active ribozyme sequences. The ribozyme can be separated into discrete enzyme and substrate strands such that the ribozyme is also active in *trans*. Minimal sequences based on the invariant core nucleotides display a range of activities that are significantly increased (∼1000-fold increase in rate) through distal interhelical interactions between ancillary sequences, that stabilise the most active conformation of the ribozyme in longer native sequences (32–34). Point mutations close to the ribozyme core can also lead to significant rate enhancements (35). Discontinuous ribozymes that are characterised by lengthy intervening sequences that separate the helical stems have also been found (36,37).

The site-specific cleavage reaction of hammerhead ribozyme takes place through an SN-2-like in-line nucleophilic attack by the 2′-oxygen group on the neighbouring 3′, 5′-phosphodiester bond of the phosphodiester linkage of the target base (C17), generating a 2′,3′-cyclic phosphate and free 5′-hydroxyl terminus. The reaction conforms to an acid-base catalytic mechanism with conserved nucleotides in the ribozyme core acting as the general base (at the N1 position of G12) with the general acid being supplied by a hydrated metal ion, associated with the 2′ hydroxyl of G8 (38–42). The core of the ribozyme adopts a compact fold and cations also contribute to the stability of the folded RNA. The hepatitis delta virus (HDV), Pistol and TS ribozymes exploit a divalent metal cation for activity (43–46). Typically for such ribozymes, cleavage rates increase from Mg^2+^< Mn^2+^< Co^2+^ in the cleavage reaction (43–46).

Despite the vast amount of information known on the SARS-CoV-2 RNA genome, no ribozymes have been identified in the genome. Here we report the identification of 39 Hammerhead like ribozyme sequences in SARS-CoV-2. The conservation of ribozyme-variant sequences within SARS-CoV-2 variants and their diversity among other coronaviruses was analyzed. We show that these Hammerhead like ribozyme sequences can cleave *in vitro* with low activity compared to the conventional Hammerhead ribozyme. We present evidence indicating that ribozyme cleavage sites align with the deletion breakpoints of sgRNAs, implying *in vivo* ribozyme activity. Furthermore, ribozyme cleavage appears to be linked to the creation of specific sgRNAs crucial for viral replication.

## Materials and methods

### Materials

The materials used in this study were obtained from the following sources. The 5′ 6-FAM labeled RNA were synthesized by Takara. DNA primers for Hammerhead-variant sequence enzyme strands *in vitro* transcription template amplification and reverse transcription were purchased from Sangon Biotech (Shanghai, China). Plasmids were purchased from Gene Script (Nanjing, China). Phanta max DNA polymerase Mix was purchased from Vazyme (Nanjing, China). dNTP (91 256 389), NTP (01 130 107), DNase I (91 260 277), RNA Inhibitor (91 290 886), SuperScript First-Strand Synthesis System for RT-PCR (2 422 007) and RNase T1 (AM2283) were purchased from Thermo Fisher Scientific. RNA Clean & Concentrator kit (R1013) was purchased from ZymoResearch. RtcB ligase (M0548S) and Quick CIP (M0525S) were purchased from NEB. T7 RNA polymerase was produced in our lab.

### Hammerhead-variant sequence searches in SARS-CoV-2 genome

The RNABOB program (http://eddylab.org/software.html) was used to search SARS-CoV-2 genome sequence data (NCBI database, accession number NC_045512.2) using the descriptor detailed in Supplementary Figure S1 for the Hammerhead-variant sequence searches; the sequences are listed in Supplementary Table S1. The secondary structure was built using information from the hammerhead ribozyme covariance model (47). The Alignment of Hammerhead-variant sequences was performed using Clustal Omega Align (https://www.ebi.ac.uk/Tools/msa/clustalo/). The alignment parameters were as follows: Sequence Type (DNA), Dealign Input Sequences (no), Output Alignment Format (clustal_num), mBed-like Clustering Guide-tree (true), mBed-like Clustering Iteration (true), Number of Combined Iterations (Values 0), Max Guide Tree Iterations (Values −1), Max HMM Iterations (Values −1), Order (Input).

### Search of variant SARS-CoV-2 genomes for Hammerhead-variant sequences

SARS-CoV-2 variant genomes sequence data were obtained from the GISAID database (https://www.gisaid.org/hcov19-variants/), including Alpha variant (EPI_ISL_11 290 133), Beta variant (EPI_ISL_11 265 109), Gamma variant (EPI_ISL_1 213 215), Delta variant (EPI_ISL_1 829 914), Lambda variant (EPI_ISL_1 160 917), Omicron variant (EPI_ISL_11 270 619). The RNABOB descriptors described above were used to search against SARS-CoV-2 variant genomes. The Hammerhead-variant sequence identities are listed in Supplementary Table S2. The Hammerhead-variant sequences in each genome were divided into groups according to their approximate locations and similar sequences. Each group of sequences were aligned by Clustal Omega method as describe above. The identities of each sequence were obtained from mview tool (https://www.ebi.ac.uk/Tools/msa/mview). The identity data were used to generate a heat map using the Tableau Public, with each column representing parallel Hammerhead-variant sequence and each row representing the identity of the corresponding sequence in each virus variant genome. The lack of a corresponding Hammerhead-variant sequence defaults to 0% identity. The schematics of Hammerhead-variant sequences distribution within variants genome were generated by SnapGene4.3.6. The result of Clustal omega alignment was displayed by Jalview 2.11.2.0 (https://www.jalview.org).

### Search of coronavirus genomes for Hammerhead-variant sequences

Coronaviruses genome sequence data were downloaded from different databases, including SASR-CoV-2 (NCBI, NC_045512.2), Bat CoV-RaTG13 (NGDC database, GWHABKP000000001), Pangolin CoV (ViPR Database, MT084071), SARS-CoV (NCBI, NC_004718.3), MERS-CoV (NCBI, NC_019843.3), PEDV (porcine epidemic diarrhoea virus) (NCBI, LT900501.1). The RNABOB descriptors described above were used to search against respective coronaviruses genome. The Hammerhead-variant sequence identities are listed in Supplementary Table S3. The distribution of Hammerhead-variant sequences in coronaviruses genome was generated by GraphPad Prism 6.01. Each Hammerhead-variant sequence in Bat CoV-RaTG13, Pangolin CoV, SARS-CoV, MERS-CoV and PEDV was compared to the Hammerhead-variant sequences in SARS-CoV-2 though clustalw 2.1. Alignment parameters as follows: Gap opening penalty (15.00), Gap extension Penalty (6.66), Delay divergent sequences (30%), DNA weight matrix (IUB), Use negative matrix OFF), Toggle output order (INPUT FILE). In each round of comparison, sequence in SARS-CoV-2 that share high identity and approximate location with the sequence in Bat CoV-RaTG13/Pangolin CoV/SARS-CoV/MERS-CoV or PEDV genome were divided into one group, and the identities of the two sequences obtained by MView 1.68 (http://desmid.github.io/mview/install). The identity data were used to generate a heat map using the Tableau Public, with each column representing parallel Hammerhead-variant sequence and each row representing the identity of the corresponding sequence in each virus genome. The lack of a corresponding Hammerhead-variant sequence defaults to 0% identity. The result of Clustal omega alignment was displayed by Jalview 2.11.2.1 (https://www.jalview.org).

### Phylogenetic relationship analysis

Sequence alignment was carried out using the Clustal Omega Alignment tool (https://www.ebi.ac.uk/Tools/msa/clustalo). The result of Clustal omega alignment was displayed by Jalview 2.11.2.1 and the phylogenetic tree was calculated though neighbour joining method.

### Synthesis and purification of oligoribonucleotides

DNA primers for Hammerhead-variant sequence enzyme strands *in vitro* transcription template amplification were purchased as synthetic oligodeoxynucleotides from Sangon Biotech, oligonucleotide sequences are shown in the supplementary information. RNA was prepared by *in vitro* transcription using T7 RNA polymerase. The reaction contained 0.4 μM dsDNA template, 40 mM Tris–HCl, 40 mM KCl, 10 mM MgCl_2_, 2.5 mM DTT, 1 mM NTP and 3000 U/ml T7 RNA polymerase at pH 8. After incubating the mixture at 42°C for 3 h, the DNA template was digested by DNase I at 37°C for 1 h. RNA transcripts were purified on 12%, 8 M urea denaturing polyacrylamide gel and eluted with 0.3 M sodium acetate at pH 5.2 with 1 mM EDTA. It was precipitated with ethanol and dissolved finally in sterile water.

### Hammerhead-variant sequences *in vitro* cleavage activity test

15 μM ribozyme and 15 μM 6-FAM-labeled substrate strands were annealed together with 50 mM TrisHCl, pH7.5, 100 mM KCl, the mixture was heated at 95°C for 1 min, cooled to room temperature for over 1 h. MgCl_2_ was then added to a final concentration of 10 mM to initiate the cleavage reaction. After incubation at 37°C for 2 h, the cleavage reactions were stopped by adding 1 volume of stop buffer (80% v/v deionized formamide, 50 mM EDTA at pH 8.0, 0.025% w/v bromophenol blue, 0.025% *w/v* xylene cyanol). Substrate and cleavage products were separated on 12% polyacrylamide/8 M urea gels. Gels were quantitated using a Gel Doc system (Bio-rad) with λ_ex_= 480 nm, λ_em_= 520 nm. The fraction of substrate cleaved was quantitated by using ImageJ 1.51j8.

### Hammerhead-variant sequences *in vitro* cleavage site mapping

To prepare RNA ladders, 2 μl 50 μM 5′ 6-FAM labeled substrate was mixed with 0.5 μl 200 μM yeast RNA and 7.5 μl 1 ˣ RNA Sequencing Buffer, heated at 50°C for 5 min, cooled to room temperature, then added 1 μl RNase T1, incubated at room temperature for 15 min. For alkaline ladder, 3 μl 50 μM 5′ 6-FAM labeled substrate was incubated with 50 mM Na_2_CO_3_-NaHCO_3_, pH 9.0 and 1 mM EDTA for 7 min at 90 °C. CoV-HHRz cleavage reaction was conducted as described above. RNA was separated on 12% polyacrylamide/8 M urea gel.

### *In vitro* cleavage of Hammerhead-variant sequences in the presence of divalent metal ions

Ribozyme (15 μM) and 6-FAM-labeled substrate strands (15 μM) were annealed together with 50 mM Tris–HCl, pH 7.5, 100 mM KCl, the mixture was heated at 95°C for 1 min, cooled to room temperature for over 1 h. MgCl_2_ or corresponding metal ion were then added to a final concentration of 10 mM to initiate the cleavage reaction. After incubation at 37°C for 16 h. Cleavage reactions were stopped as described above and substrate and cleavage products were separated on 12% polyacrylamide/8 M urea gels. Gel images were taken and quantitated as described above.

### Single-turnover kinetics

For Hammerhead-variant sequences kinetic experiments, under single-turnover conditions, 100 μM enzyme strands and 10 μM 6-FAM-labeled substrate strands were annealed together with 50 mM Tris–HCl, pH 7.5, 100 mM KCl, the mixture was heated at 95°C for 1 min, cooled to room temperature for over 1 h. The cleavage reaction was initiated by adding magnesium or manganese with 10 mM final concentration. At each time point, the cleavage reactions were stopped by adding 1 volume of stop buffer (80% deionized formamide, 50 mM EDTA at pH 8.0, 0.025% bromophenol blue, 0.025% xylene cyanol). Substrate and cleavage products were separated on 12% polyacrylamide/8 M urea gels, and the fraction of substrate cleaved was quantitated by using Gel Doc system (Bio-rad) and ImageJ 1.51j8 software. The first order rate constants (*k*_obs_) for HH16, CoV-HHRz and mutant sequences in were calculated by plotting the fraction of substrate cleaved (*f_t_*) versus time (*t*) and fitting to the equation *f_t_* = 1 – exp(*k*_obs_*t*) with GraphPad Prism 6.01.

### Detection of the Hammerhead-variant sequence cleavage product 2′,3′-cyclic phosphate

For HH16-S and CoV-HHRz-27665, 400 μM enzyme strands and 400 μM 6-FAM-labeled substrate strands were annealed in the presence of 50 mM Tris–HCl, pH 7.5, 100 mM KCl, the mixture was heated at 95°C for 1 min, cooled to room temperature for over 1 h. Magnesium or manganese were then added to a final concentration of 10 mM to initiate the cleavage reaction (200 μl volume). After incubation at 37 °C at an appropriate time (HH16 for 10min, CoV-27665 with wild type enzyme strand for 16 h, and CoV-HHRz-27665 with mutant enzyme strand for 10 min), the cleavage reaction products were ethanol precipitated at −80 °C for over 1 h by adding 600 μl 100% ethanol and 20 μl 3 M sodium acetate at pH 5.2.

A terminal cyclic 2′,3′-phosphate is inert towards CIP (calf intestinal alkaline phosphatase) treatment, acid hydrolysis generates a 3′-phosphate that is a substrate for CIP and enables detection of the cyclic 2′,3′-phosphate. For the acid treatment, the RNA pellet was dissolved with 45 μl water and 5 μl 100 mM HCl was added and followed by incubation on ice for 3 h and ethanol precipitation. For subsequent CIP treatment, the RNA pellet was dissolved with 15 μl water and added 2 μl 10× CutSmart buffer, 0.2 μl RNase Inhibitor (40 U/μl) and 1 μl CIP in total volume of 20 μl followed by incubation at 37 °C for 20 min. 80 μl water was added before ethanol precipitation. The RNA pellet was dissolved with 15 μl water and concentration was measured using nanodrop spectrometer, about 300 ng treated cleavage products for each group were loaded on 20% polyacrylamide/8 M urea gel and detection was made with Gel Doc system (Bio-rad).

### Mapping of the breakpoint of sgRNA in SARS-CoV-2 transcriptome data

SARS-CoV-2 transcriptome sequencing data were obtained from eight studies as follows: Kim *et al.* (https://doi.org/10.17605/OSF.IO/8F6N9) (15), Finkel *et al.* (GSE149973) (8), Blanco-Melo *et al.* (SRR11517741, SRR11517855-SRR11517858) (12), Ranasinghe *et al.* (16), Davidson et.al (zendo 3 722 580) (13), Taiaroa *et al.* (SRR11267570) (18), Suzuki *et al.* (SRR11811022) (17), Emanuel *et al.* (SRR11550047, SRR11550045) (14). Among these studies, Kim *et al.* (15) and Finkel *et al.* (8) provided the breakpoints as well as count number of corresponding subgenomic RNA in SARS-CoV-2 infected cell with processed RNA sequencing data. The analysis results are available in corresponding reference supplementary tables (8,15).

For the other six studies, the raw sequencing data in fastq format were downloaded from the corresponding databases. The long-read data from nanopore sequencing was aligned to the SARS-CoV-2 genome (NC_045512.2) by ngmlr (v.0.2.7), and all data from each run was merged with samtools (v1.8) followed by using sniffles (v1.0.11) to identify potential breakpoints. For the next generation sequencing data, both single-end and paired-end reads were aligned to SARS-CoV-2 genome by STAR (v2.7.0d) followed by using the lumpy (v 0.2.13) to identify the potential breakpoints. Analysis results are displayed in Supplementary Table S4.

### *In vivo* and *in vitro* SHAPE reactivity of HHRz Hammerhead-variant sequences analysis

*In vivo* and *in vitro* SARS-CoV-2 genome wide SHAPE reactivity data was obtained from the studies of Manfredonia et al. (48) and Sun *et al.* (49). The SHAPE reactivities of the corresponding Hammerhead-variant sequences were selected. The example of the SHAPE reactivity *in vivo* and *in vitro* for Hammerhead-variant sequence was plotted as histogram by using GraphPad Prism 6.01 and displayed as Supplementary Figure S4.

### Generation of HHRz fragment RNA with HDV/HHRz construction

To generate the CoV-HHR sequence with 5′ fragment bearing a 2′,3′-cyclic phosphate (CoV-HHRz > P), a plasmid (Gene Script, Nanjing, China) was constructed consisting a CoV-HHR sequence 5′ fragment followed by the sequence of a mutant form of the hepatitis delta virus (HDVr) antigenomic ribozyme sequence (CoV-HHRz-HDVr). Double stranded DNA template was obtained by PCR using a forward primer that containing T7 promoter and reverse primer. A control template lacking the HDV ribozyme sequence was used to generate CoV-HHRz bearing a 3′-OH.

To generate the CoV-HHRz sequence 3′ fragment bearing a 5′-OH (OH-CoV-HHRz), a plasmid (Gene Script, Nanjing, China) was constructed containing an engineered hammerhead ribozyme (HHr) sequence followed by CoV-HHRz sequence 3′ fragment. Double stranded DNA template was obtained by PCR using forward primer that containing T7 promoter and reverse primer. A control template lacking the engineered HHr sequence was used to generate CoV-HHRz bearing a 5′- triphosphate. The HDVr, HHr ribozyme sequences, CoV-HHRz 5′ fragment and CoV-HHRz 3′ fragment, primer sequences for plasmid PCR are displayed in Supplementary information.

### Ligation assay of the CoV-HHRz related subgenomic RNA junction flanking sequences

*In vitro* transcribed CoV-HHRz 5′ fragment and CoV-HHRz 3′ fragment RNA was first splinted with a DNA oligonucleotide (∼ 20 μM final concentration for each RNA and DNA oligo in final reaction volume 13 μl). The RNA/DNA oligo mixture was then incubated at 65°C for 5 minutes, 25°C for 3 minutes, and then kept at 4°C for approximately 10 minutes before being added to the ligation reaction. Next, 5′/3′ fragment RNA ligation reactions (20 μl final volume) containing 2 μl 10× RtcB Reaction Buffer, 0.1 mM final concentration GTP, 1 mM final concentration MnCl_2_, and 1 μl RtcB RNA ligase were incubated at 37°C for 1 h. The reactions were concentrated using an RNA Clean & Concentrator kit (ZymoResearch), which included an on-column DNase I (Thermo Fisher Scientific) digestion. RNA was eluted with 10 μl each of water. Following ligation reactions, cDNAs were prepared using 7 μl of concentrated RNA with the SuperScript First-Strand Synthesis System for RT-PCR using 5′ end 6-FAM labelled DNA reverse transcription primer. Reverse transcription (RT) reactions were incubated at 65°C for 5 min then 2 min on ice, followed by incubation at 55°C for 90 min. The reverse transcription products were detected by capillary sequencing (Saiyin Biotech (Shanghai, China)).

## Results

### Bioinformatic searches for Hammerhead ribozyme candidate sequence in SARS-CoV-2 sequence

**Figure 1.**
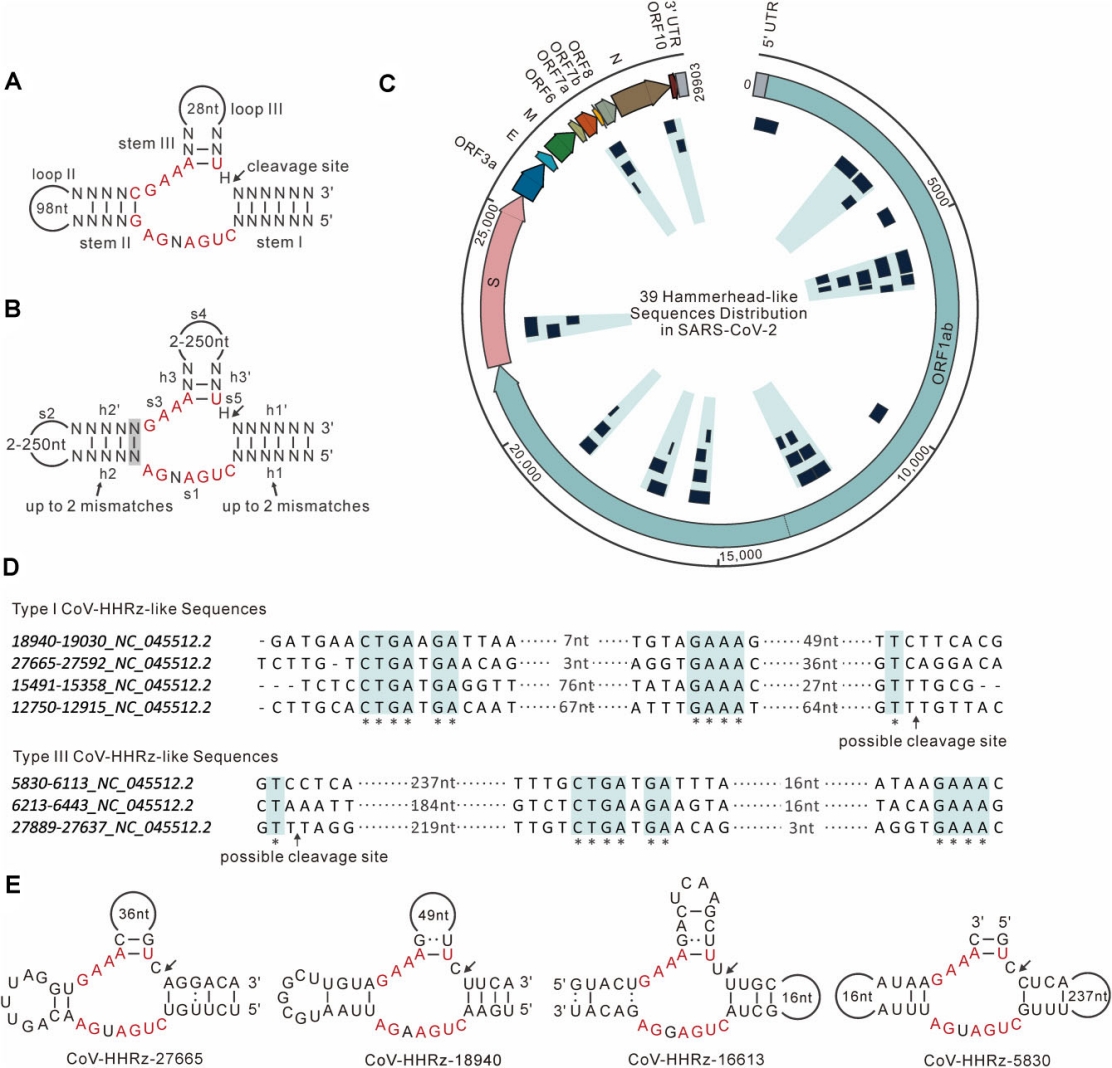
Identification of Hammerhead-variant sequences in the SARS-CoV-2 genome. (**A**) Covariance model of hammerhead ribozyme. Conserved nucleotides are highlighted in red. The arrowhead indicates the cleavage site. (**B**) RNABOB search criteria for Type I Hammerhead-variant sequences in this study. The eleven conserved bases are highlighted in red. The grey box shows relaxed base pair adjacent to the catalytic core. Up to two mismatches are allowed in stem I and II. Loop II and III are allowed to be up to 250nt. (**C**) Distribution of Hammerhead-variant sequences in SARS-CoV-2 genome. The outer circle indicates the SARS-Cov2 genome location (nt), ORFs and 5′ and 3′ UTRs. The light blue radial shadows indicate the cluster of the sequences for the location and coverage of the 39 Hammerhead-variant sequences (as inset navy-blue boxes) in SARS-Cov2 genome. (**D**) Sequence examples alignment of Hammerhead-variant sequences. The accession numbers and location numbers are shown. Conserved nucleotides are highlighted by blue shading and asterisks. The length of loop II and III are shown. (**E**) Examples of folded Hammerhead-variant sequences.

The SARS-CoV-2 sequence is available in the NCBI database (50) (Accession number NC_045512.2). The SARS-CoV-2 genome sequence was searched for the established self-cleaving ribozymes: Twister, HDV, GlmS, Hairpin and Hammerhead etc. (51) using ribozyme specific criteria with RNABOB (http://eddylab.org/software.html), only hammerhead ribozyme sequences were identified in this search. The catalytic center of hammerhead ribozyme sequences consists typically of 13 highly conserved nucleotides that form a three-way junction with stem I/II/III (52). Hammerhead ribozymes self-cleave at 3′ end of the sequence NUH (N can be any of the four nucleotides and H can be A U or C) (Figure 1A) (47). The search criteria were based on the known hammerhead ribozyme sequence domains, allowing up to two mismatches in the stem I and II and 250 nucleotides in the loops. The G-C base pair in stem II was less stringent (N-N) in our search since there are reports that consider the catalytic core to consist of 11 conserved nucleotides (Figure 1B) (29). The exemplar syntax for the hammerhead ribozyme type I is shown (Supplementary Figure S1). A total of 39 Hammerhead-variant sequences (CoV-HHRz) were identified in SARS-CoV-2 (Supplementary Table S1). There are 12 type I, 15 type II and 12 type III CoV-HHRz sequences (Supplementary Table S1). The CoV-HHRz sequences are widely distributed along the SARS-CoV-2 genome with preference for ORF 1ab (Figure 1C). The CoV-HHRz sequences are present in the genome as clusters rather than evenly scattered (Figure 1C). Examples of the type I CoV-HHRz sequences (Figure 1D) and secondary structure folding (Figure 1E) are displayed.

The probability of the Hammerhead ribozyme core sequence occurring at random in SARS-CoV-2 genome was estimated to be 7.6 × 10^−7^, while the probability of the Hammerhead ribozyme core sequence occurring at a 3-way junction was estimated to be 7.5 × 10^−3^ (Supplementary Information andSupplementary Document 1. 1), suggesting that the probability that Hammerhead ribozyme-variant sequences are present in SARS-CoV-2 genome by random chance is relatively low.

### Activity of Hammerhead like ribozyme candidate sequences in SARS-CoV-2 *in vitro*

**Figure 2.**
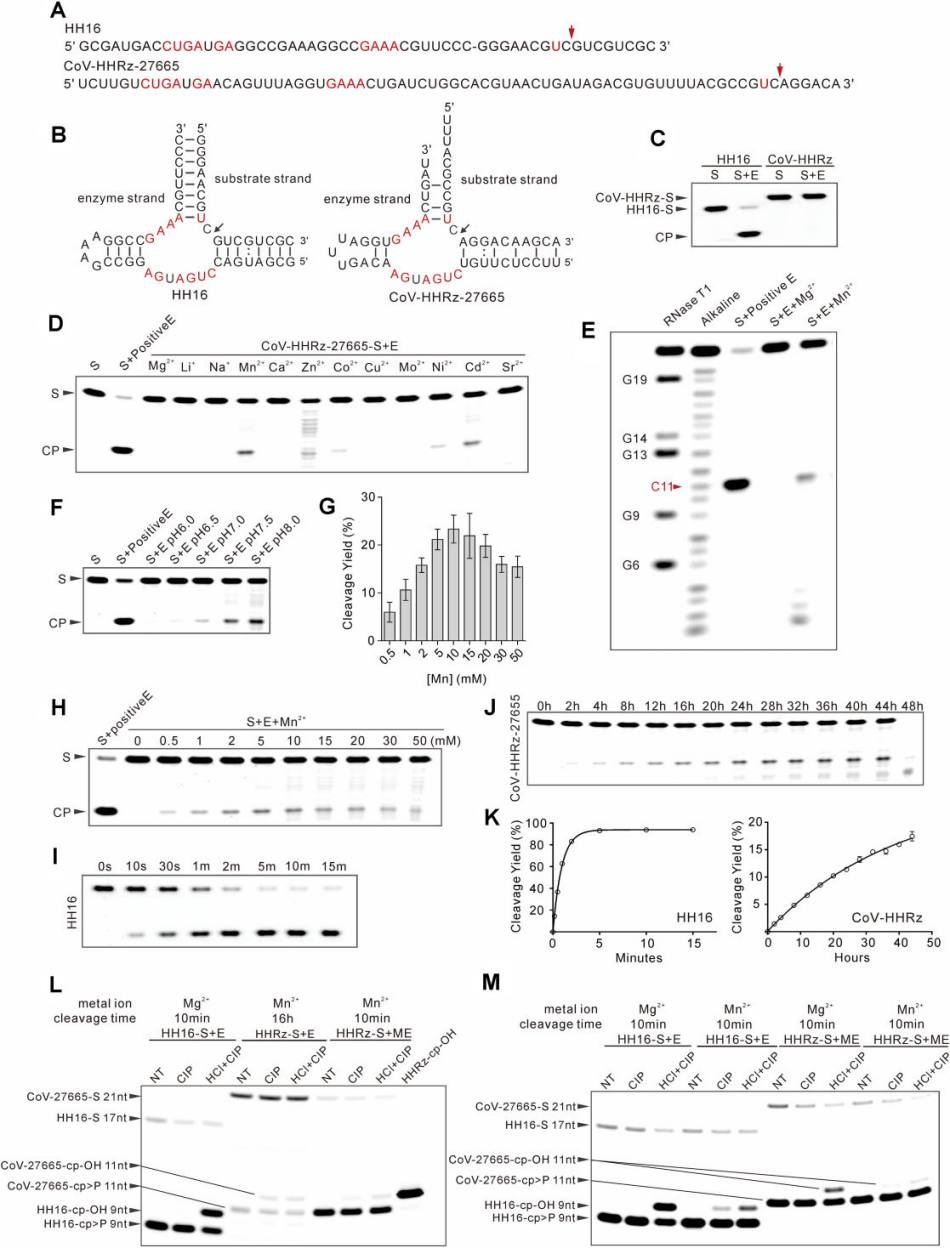
*In vitro* activity of Hammerhead-variant sequences in the SARS-CoV-2 genome. (**A**) Sequences of the conventional hammerhead ribozyme HH16 and the Hammerhead-variant sequence (CoV-HHRz-27665). Conserved nucleotides are marked in red. The arrowhead indicates the cleavage site. (**B**) Secondary structures of the conventional hammerhead ribozyme and CoV-HHRz-27665 substrate and enzyme strands. (**C**) Tests of the cleavage activity of CoV-HHRz-27665, compared to HH16. 5′ 6-FAM-labeled substrate strand (S) and purified enzyme strand (E) were mixed under cleavage conditions at 37 °C for 2 h. All subsequent cleavage products were resolved on 12% denaturing polyacrylamide gels. (**D**) CoV-HHRz-27665 activity in the presence of metal ions. Samples were incubated for 16 h in the presence of 10 mM of the indicated ion. The active CoV-HHRz-27665 mutant 2 ((positive E), Supplementary Figure S2A) with the CoV-HHRz-27665 (S) was used as a positive control (positive E) throughout. (**E**) Mapping the cleavage site of CoV-HHRz-27665. Labeled substrate (S) was partially digested with RNase T1 (G nucleotides are marked) or with alkali (OH^−^) or was incubated with enzyme (E) RNA with Mg^2+^/Mn^2+^, as previously described. C11 is highlighted in red. (**F**) pH dependence of CoV-HHRz-27665 activity. (**G**) Quantitation of Mn^2+^ dependence by % yield. (**H**) CoV-HHRz-27665 activity on titration of Mn^2+^, 5′-labeled substrate RNA was incubated with enzyme RNA for 16 h as indicated. (**I**) HH16 cleavage kinetics *in vitro*; samples were incubated in cleavage buffer with 10 mM Mg^2+^ at 37 °C for the given times (t). (**J**) Cleavage kinetics of CoV-HHRz-27665 as for (Figure 4I) in the presence of 10 mM Mn^2+^. (**K**) Plots of HH16 and CoV-HHRz-27665 taken from (I) and (J), the first order rate constants (*k*_obs_) of hammerhead ribozyme or Hammerhead-variant sequence were calculated as described in the methods section. Error bars are the standard deviation of 3 independent experiments. (**L**) Detection of the 2′,3′-cyclic phosphate of CoV-HHRz-27665 5′ cleavage product. HH16 as positive control. (**M**) Comparison of hammerhead ribozyme cleavage product 3′ end 2′,3′-cyclic phosphate with HCl or HCl + CIP treatment in the presence of Mg^2+^ or Mn^2+^.

The SARS-CoV-2 Hammerhead-variant sequences appear to have the potential to fold into an active ribozyme. To assess activity of the 39 CoV-HHRz sequences *in vitro*, the putative ribozyme sequences were separated into the enzyme and substrate strands. The enzyme strand was made by *in vitro* transcription and the substrate was chemically synthesized with a 6-FAM at the 5′ end. The enzyme and the substrate strands were incubated together under standard conditions (50 mM Tris–HCl, pH 7.5, 100 mM KCl, 10 mM Mg^2+^) in which conventional hammerhead ribozymes are active and visualized by gel electrophoresis. No cleavage was detected with these CoV-HHRz sequences under standard conditions (data not shown). The activity of these CoV-HHRz sequences may be dependent on the combination of multiple factors such as pH, cation, time or temperature. Under the standard conditions with Mg^2+^, the CoV-HHRz-27665 sequence is inactive, compared to the well characterised, active Hammerhead ribozyme (HH16) (53) (Figure 2A–C). The cleavage activity of the CoV-HHRz-27655 was further tested with a variety of different cations and conditions. In the presence of the transition metal cations Mn^2+^, Co^2+^ or Cd^2+^, a cleaved substrate was detected (Figure 2D). To examine whether the cleavage site was the same as the conventional Hammerhead ribozyme, a CoV-HHRz-27655 mutation was designed as a positive control containing stem II and loop II of SM-HHRz and complete base paired stem I and III, retaining an unchanged SARS-CoV-2 substrate sequence (Supplementary Figure S2A, mutant 2) (39). The alkaline ladder and the cleavage of the CoV-HHRz-27655 mutation together showed the cleavage site to be the same as a typical Hammerhead ribozyme (Figure 2E). The pH dependence of the ribozyme was investigated and activity observed with maximal cleavage at pH 8.0, consistent with a general acid-base catalytic mechanism of self-cleavage (Figure 2F). Titration of Mn^2+^, resulted in a progressive increase in cleavage product with maximum cleavage at ∼ 10 mM Mn^2+^ (Figure 2H, quantified in Figure 2G). In the presence of 10mM Mn^2+^, CoV-HHRz-27665 displayed slow ribozyme kinetics with a rate of 0.025/h (Figure 2J and K), compared to the ribozyme control (HH16) in the presence of Mg^2+^ (Figure 2I and K). Comparison of the end-products of CoV-HHRz-27655 and the HH16 control in the presence of Mn^2+^, after treatment with HCl and CIP (54), suggests that self-cleavage generates 2′,3′-cyclic phosphate and free 5′-hydroxyl terminus ends (Figure 2L and M). Collectively, these results suggest that the ribozyme adopts a general acid-base cleavage mechanism.

Two sets of chimeric RNAs were made for CoV-HHRz-27665 and CoV-HHRz-5830; Mutant 1 was designed to retain stem loop II to introduce full base-pairing to stems I, II and III, while mutant 2 was based on mutant 1 with stem-loop II replaced by that of the conventional SM-HHRz (Supplementary Figure S2A). For the CoV-HHRz-27665 and 5830 mutants 1 and 2, time courses were used to measure ribozyme kinetics in the presence of Mg^2+^ or Mn^2+^, in comparison with a HHRz ribozyme control. Plots of cleavage versus time produce the ribozyme cleavage rates shown in Supplementary Figure S2B and S2C and tabulated in Supplementary Figure S2E. The wild type sequence CoV-HHRz-5830 is inactive under both the standard buffer condition with Mg^2+^ or Mn^2+^ (Supplementary Figure S2C). In the presence of Mg^2+^, CoV-HHRz-27665 and 5830 mutants 1 and 2 become active ribozymes compared to the original CoV-HHRz RNAs that are inactive under these conditions, and mutant 2 is more active than mutant 1 (Supplementary Figure S2E). Moreover, in the presence of Mn^2+^, CoV-HHRz-27665 and 5830 mutants 1 and 2 have rate constants that are much faster than the wild-type CoV-HHRz RNAs. Specifically, for the CoV-HHRz-27665 mutant 2, in the presence of 10 mM Mn^2+^, cleavage was complete within 10 seconds which was too fast for accurate rate measurements under these reaction conditions (not shown). To obtain a measurable rate constant for CoV-HHRz-27665 mutant 2 in the presence of Mn^2+^, the Mn^2+^ concentration was reduced and kinetics experiments preformed at 0.1, 0.2, 0.3, 0.4, 0.5, 1 and 2 mM Mn^2+^. The rate constant of CoV-HHRz-27665 mutant 2 in the presence of 10mM Mn^2+^ was extrapolated to be ∼1204 min^−1^, close to that of the control HH16 (34) (Supplementary Figure S2E). The extrapolation from the kinetic measurements in the presence of lower [Mn^2+^] is shown in Supplementary Figure S2D. Note that the CoV-HHRz 5830 wild-type sequence is inactive in the presence of Mg^2+^ or Mn^2+^ (Supplementary Figure S2C), however, both mutants 1 and 2 of the 5830-sequence become more active. Mutations to CoV-HHRz 6072 and 12 312 were generated and their ribozyme activity examined under the same conditions. Analogously for each sequence, mutant 2 is active in the presence of Mn^2+^ while mutant 1 and the wild type RNA are inactive with or without Mg^2+^ or Mn^2+^ (Supplementary Figure S3). Thus, these results further support the notion that the CoV-HHRz sequences retain characteristics of a typical hammerhead ribozyme for catalysis.

### Hammerhead-variant sequences cleavage sites coincide with the SARS-CoV-2 sgRNA breakpoints

**Figure 3.**
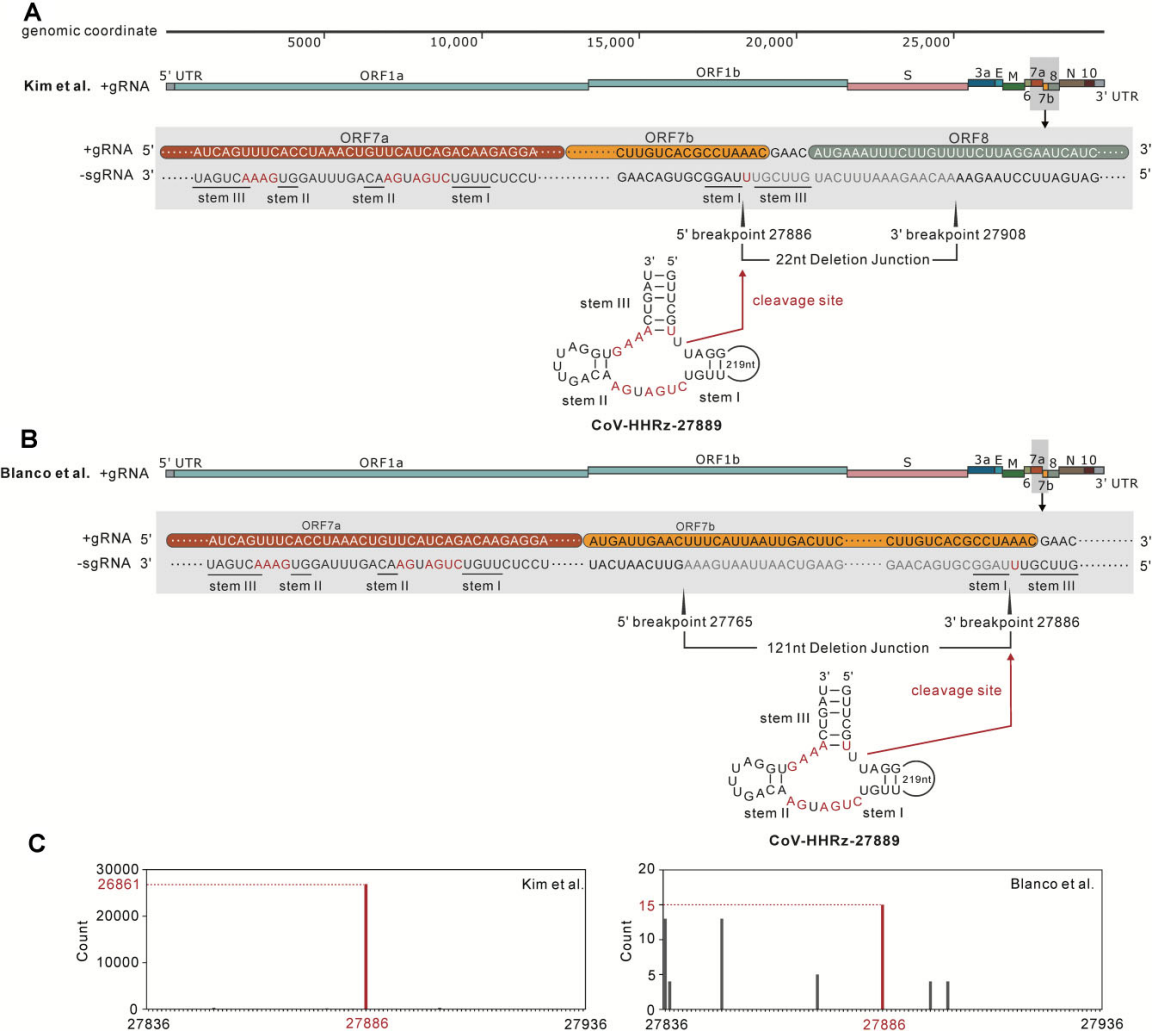
Hammerhead-variant sequences cleavage sites coincide with the SARS-CoV-2 sgRNA breakpoints. (**A**) The location of CoV-HHRz-27889 and the breakpoints of the subgenomic RNA identified by Kim and colleagues (15). The schematic shows the organisation of the SARS-CoV-2 genomic RNA including 5′ and 3′ untranslated regions (UTRs) and open reading frames (ORFs), aligned against the whole SARS-CoV-2 genome. The magnified grey shaded region highlights the position of CoV-HHRz-27889 which spans ORF7a (in red) and ORF8 (blue grey) of the subgenomic RNA. The 5′ and 3′ breakpoints of the subgenomic RNA are marked by elongated black triangles, the deleted sgRNA junction fragment sequence is shown in light grey type. CoV-HHRz-27889 is located on the negative strand, and the stems of the CoV-HHRz are marked. Conserved bases are highlighted in red. The red arrow indicates the cleavage site, and the deleted nucleotides are represented in grey. (**B**) The location of CoV-HHRz-27889 and the relationship to breakpoints of the subgenomic RNA identified by Blanco and colleagues (12). The magnified grey shaded region highlights the position of CoV-HHRz-27889 which spans ORF7a (in red) and OR7b (orange) of the subgenomic RNA. The sequences, 5′ and 3′ breakpoints, locations of CoV-HHRz stems, cleavage site, conserved nucleotides, and the deleted junction fragment are as described above. (**C**) The histograms display the counts for the detected 27 886 (red) breakpoints compared to the neighbouring ±50 nucleotides. The nucleotide position for each breakpoint is represented by a red bar, and the counts denoted in red and by red dotted reference lines.

**Figure 4.**
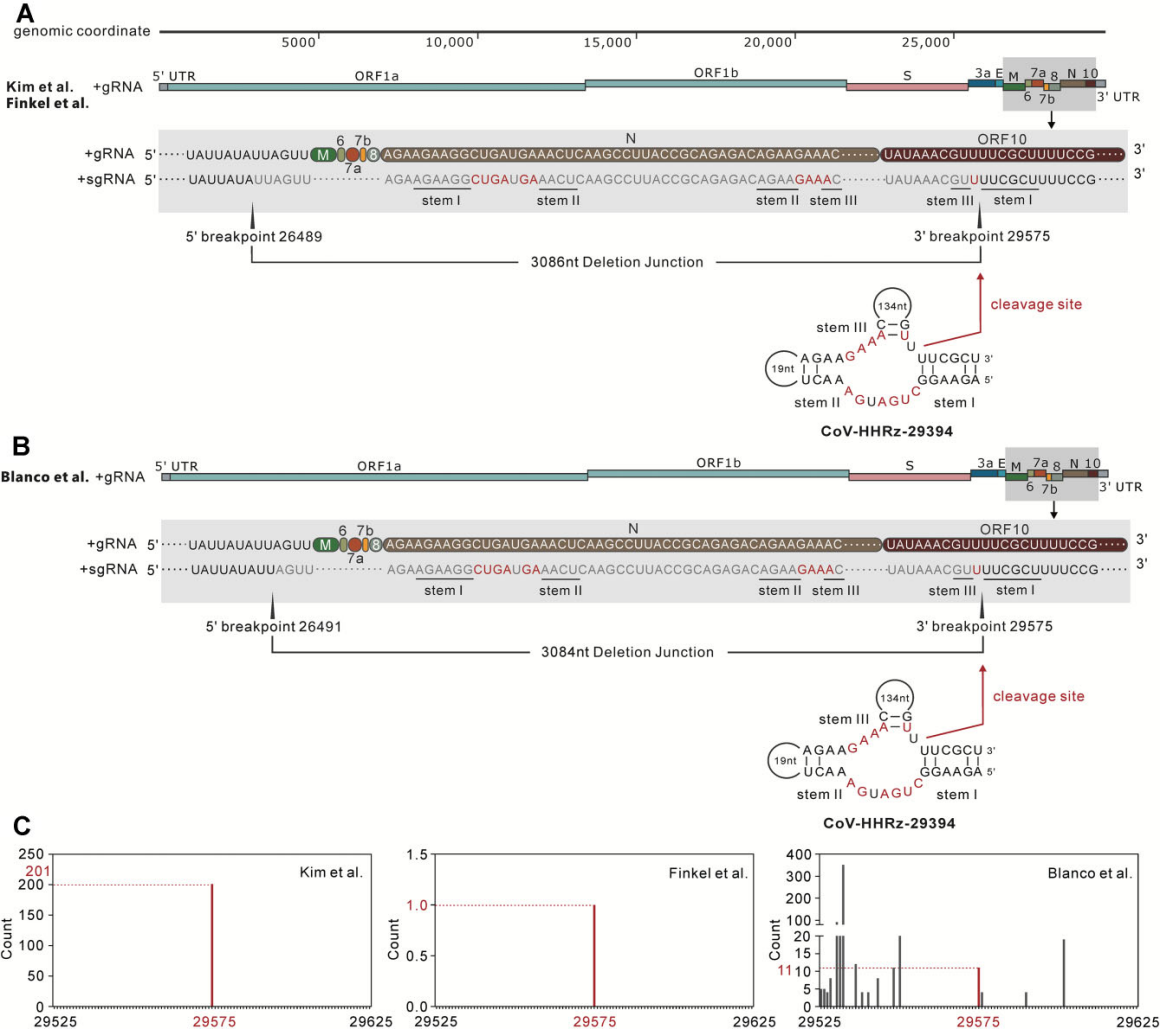
Hammerhead-variant sequences cleavage sites coincide with the SARS-CoV-2 sgRNA breakpoints (continued). (**A**) The location of CoV-HHRz-29394 and the breakpoints of the subgenomic RNA identified by Kim, Finkel and colleagues (8,15) is a point of further interest. The organisation of the SARS-CoV-2 genomic RNA including 5′ and 3′ untranslated regions (UTRs) and open reading frames (ORFs), aligned against the whole SARS-CoV-2 genome. The magnified grey shaded region highlights the position of CoV-HHRz-29394, spanning ORFN (in dark grey) and ORF10 (brown) of the subgenomic RNA. The 5′ and 3′ breakpoints of the subgenomic RNA are marked by elongated black triangles, the deleted sgRNA junction fragment sequence is shown in light grey type. CoV-HHRz-27889 is located on the positive strand, and the stems of the CoV-HHRz are marked. Conserved bases are highlighted in red. The red arrow indicates the cleavage site, and the deleted nucleotides are represented in grey. (**B**) The location of CoV-HHRz-29394 and the relationship to breakpoints of the subgenomic RNA identified by Blanco and colleagues (12) should also be considered. The magnified grey shaded region highlights the position of CoV-HHRz-29394 which spans ORFN (in dark grey) and ORF10 (brown) of the subgenomic RNA. The sequences, 3′ breakpoints, locations of CoV-HHRz stems, cleavage site, conserved nucleotides, are as described above. The the deleted junction fragment and 5′ breakpoint differs from that of Kim and colleagues (15) by 2 nucleotides. (**C**) The histograms display the counts for the detected 29 575 (red) breakpoints compared to the neighbouring ±50 nucleotides. The nucleotide position for each breakpoint is represented by a red bar, and the counts denoted in red and by red dotted reference lines.

SARS-CoV-2 transcriptome has been generated by several labs using different sequencing strategies, different host systems and viral isolates (8,12–18). The SARS-CoV-2 transcriptome data revealed that the expressed sgRNAs contain sequences with non-canonical junctions or deletion sequences (15). To investigate if the CoV-HHRz ribozyme self-cleaves at the predicted cleavage site *in vivo* and whether the cleavage site coincides with the deletion point of the expressed sgRNA, SARS-CoV-2 transcriptome sequences from several independent labs were used to map the deletion breakpoint of the expressed sgRNAs and their association with the ribozyme cleavage site. The identification of deletion breakpoints by bioinformatic methods is described in detail in the materials and method section, the results are displayed in Supplementary Table S4. The count numbers of each specific breakpoint are calculated and displayed in Figures 3C, 4C and Supplementary Figure S4C. The cleavage site of the four CoV-HHRz sequences do indeed coincide with the breakpoint of the expressed sgRNAs in SARS-CoV-2 transcripts (Tables 1 and 2). Although Kim and colleagues and Blanco and colleagues (12,15) used different cell lines (vero cells vs human lung alveolar cells) and sequencing strategies (Nanopore direct RNA sequencing vs Illumina NGS sequencing), they independently identified the deletion breakpoint 27 886 in the expressed sgRNAs using virus from the same Pango lineage. In our searches, the deletion breakpoint 27 886 was shown to coincide with the cleavage site of the CoV-HHRz-27889 ribozyme (Figure 3A). The cleavage site of the ribozyme is within ORF 7b and matches the 5′ breakpoint of sgRNA transcript in the Kim study and the 3′ breakpoint of another sgRNA transcript in Blanco study (Figure 3A and B). It should be noted that the count number for the breakpoint 27 886 is high (26 861 counts) compared to the neighbouring sites (Figure 3C). The cleavage site of the ribozyme and the 5′ and 3′ breakpoints of the sgRNA transcripts in the Kim and Blanco studies are shown (Figure 3A and B), indicating that the breakpoints (12,15) are a record of ribozyme cleavage *in vivo*. Kim, Finkel, Blanco and colleagues (8,12,15) have independently identified the same deletion breakpoint 29 575 (located in ORF10) in the expressed sgRNAs within different cell lines or in viruses from different Pango lineages by different sequencing strategies (Tables 1 and 2). The position of the cleavage site of the ribozyme and the 5′ and 3′ breakpoints of sgRNAs transcripts in the Kim, Finkel and Blanco studies are graphically shown in Figure 4A and B, and are consistent with the identification of a genuine breakpoint produced by the cleavage of the ribozyme *in vivo*. It is noteworthy that the Kim and Finkel studies consistently identified the breakpoint 29 575 in the same sgRNA and Blanco *et al.* found the same the breakpoint 29 575 in an additional sgRNA (Figure 4C and Table 2). In addition, the Blanco study showed that the breakpoint 29 575 was absent in samples at 5 hours of viral infection but present at 24 hours, suggesting that *in vivo* activity of the ribozyme may be associated with the life cycle of the virus and the stage of viral infection. A further two breakpoints (16 633 and 17 024 located in ORF1ab nsp13) were identified by Ranasinghe *et al.* using a more recent viral Pango lineage (Delta B.1.617.2) from clinical sputum and nasopharyngeal swabs (16). The breakpoints 16 633 and 17 024 also match the predicted cleavage sites of HHRz-16613 and 16 652 ribozymes with the count number (Supplementary Figures S4A–C). Overall, the identification of ribozyme cleavage sites within identical breakpoint sgRNA sequences, as confirmed by four distinct laboratory studies across various cells and virus types, conducted under different experimental conditions and utilizing independent sequencing techniques, is indicative of *in vivo* ribozyme cleavage playing a functional role in the sgRNA processing of ORF7b, ORF10 and ORF1ab nsp13 RNAs, critical for viral packaging and proliferation.

**Table 1.** Information on RNA sequencing data

Sequencing data							
References	SARS-CoV-2 strain	EPI_ISL ID	Pango lineage	Cell/sample source	Infection time	Sequencing platform	Data accession
Kim et al.	BetaCoV/Korea/KCDC03/2020	EPI_ISL_407 193	A (Pango v.4.3 PANGO-v1.20)	Vero cells (ATCC, CCL-81)	24h	Nanopore direct RNA sequencing	DOI 10.17605/OSF.IO/8F6N9
Ranasinghe et al.	sputum/nasopharyngeal swab samples		Delta B.1.617.2	sputum/nasopharyngeal swab samples	–	Nanopore direct RNA sequencing	–
Finkel et al.	BetaCoV/Germany/BavPat1/2020	EPI_ISL_406 862	B.1 (Pango v.4.3 PANGO-v1.20)	Vero E6	5 h	Illumina Nextseq 500	SRR11713355
					24 h	Illumina Nextseq 500	SRR11713363
Blanco et al.	USA-WA1/2020 (NR-52281)	EPI_ISL_404895.2	A (Pango v.4.3 PANGO-v1.20)	Huam lung alveolar cell A549-ACE2	24 h	Illumina NextSeq 500	SRR11517741
				Naive ferrets nasal washes and trachea	72 h	Illumina NextSeq 500	SRR11517855-SRR11517858

Detection of cleaved Hammerhead ribozymes in SARS-CoV-2 samples from four independent laboratories and studies.

RNA sequence data sources are given including; Publication, Strain, Source ID, Lineage, Sample origin, Infection time, Detection of cleaved Hammerhead ribozyme, sequencing platform and Accession numbers.

**Table 2.** Location of active Hammerhead Ribozyme cleavage sites in SARS-CoV-2 subgenomic RNAs

Sequencing data			Subgenomic RNA junctions			HHRz					
References	Cell/sample source	Infection time	5_breakpoint	3_breakpoint	Count	Junction length	HHRz ID	Start	End	Cleavage site	Matched breakpoint
Kim et al.	Vero cells	24 h	27 886	27 908	26 861	22nt	HHRz-27 889	27 637	27 889	27 886	5_breakpoint
		24 h	26 489	29 575	201	3086nt	HHRz-29 394	29 394	29 581	29 575	3_breakpoint
Ranasinghe et al.	sputum/nasopharyngeal swab samples	–	4027	16 633	9	12606nt	HHRz-16 613	16 613	16 669	16 633	3_breakpoint
		–	13 548	17 024	3	3476nt	HHRz-16 652	16 652	17 030	17 024	3_breakpoint
Finkel et al.	Vero E6	5 h	–	–	–	–	–	–	—	—	—
		24 h	26 489	29 575	1	3086nt	HHRz-29 394	29 394	29 581	29 575	3_breakpoint
Blanco et al.	Huam lung alveolar cell A549-ACE2	24 h	27 765	27 886	15	121nt	HHRz-27 889	27 637	27 889	27 886	3_breakpoint
		24 h	26 491	29 575	11	3084nt	HHRz-29 394	29 394	29 581	29 575	3_breakpoint
	Naive ferrets nasal washes and trachea	72 h	–	–	–	–	–	–	–	–	–

Information on screened out CoV-HHRz and corresponding subgenomic RNA

The positions and IDs within the subgenomic RNAs of the ribozyme sequences, cleavage sites and related ORFs. Note that HHRz-27889 has been identified by two different labs (Kim and Blanco) and HHRz-29394 has been identified by three different labs (Kim, Finkel and Blanco).

Genome-wide chemical probing of the structure of SARS-CoV-2 RNA *in vitro* and *in vivo* is available (48,49,55–57). The folding of the four hammerhead ribozymes from the published data was further investigated. Chemical probing of the RNA by selective-2-hydroxyl acylation analysed by primer extension (SHAPE) analysis and dimethyl sulphate (DMS) of CoV-HHRz-16613 shows that the folding of the short RNA (57 nucleotides) is consistent with the presence of a structured RNA with regions of low SHAPE and DMS reactivity corresponding to stems I-III and higher reactivity in loop sequences, compatible with a possible hammerhead three-way junction (Supplementary Figure S4D) (48,58). However, other longer CoV-HHRz RNA sequences were only partially consistent with hammerhead ribozyme formation (not shown) probably due to the presence of long intervening sequences between ribozyme stem sequences; correct folding of these sequences may require specific conditions, or may not be possible.

In order to provide biochemical evidence that cleavage of CoV-HHRz-27889 may contribute to sgRNA processing, the products of ribozyme cleavage were assessed for RNA ligation *in vitro* using the human ligase RtcB that has been shown to join fragments with a 2′,3′-cyclic phosphate end to 5′-OH ending RNA fragments (59). The 5′ fragment of CoV-HHRz-27889 and the 3′ end RNA fragment (Supplementary Figure S5A) was prepared by using constructs derived from HDV or HHr ribozymes. The 5′ cleavage fragment of CoV-HHRz-27889 terminates with a 2′,3′-cyclic phosphate (CoV-HHRz > P) at its 3′ end and was generated by HDV cleavage, the 3′end RNA fragment containing a 5′-OH (OH-CoV-HHRz) was made through Hammerhead ribozyme cleavage (Supplementary Figure S5A). The control experiments combined RNA molecules with 3′ end phosphates (CoV-HHRz > P) and 5′ end phosphates (P-CoV-HHRz), 3′ end hydroxyl (CoV-HHRz-OH) with 5′ end phosphate (P-CoV-HHRz), and 3′ end hydroxyl (CoV-HHRz-OH) with 5′ end hydroxyl groups (OH-CoV-HHRz). Pairs of the prepared purified fragments including controls were incubated with a DNA splint in the presence of RtcB ligase (59), reverse transcribed and analysed by gel electrophoresis (Supplementary Figure S5B), followed by capillary gel sequence analysis (Supplementary Figure S5A, B and S5C–F). The ligation reaction of the 5′ cleavage fragment of CoV-HHRz-27889 that has a 2′,3′-cyclic phosphate (CoV-HHRz > P) with the 5′-OH (OH-CoV-HHRz) fragment yields a ligation product that was detected by gel electrophoresis, and capillary sequencing confirmed the presence of a full-length ligation product (Supplementary Figure S5A–C (lower panel)). In contrast, no ligation product was detected for the control samples lacking the DNA splint or RtcB ligase (Supplementary Figure S5C (upper panels)). Similarly, no ligation product was detected for the fragments that combined 5′ and 3′ phosphate and hydroxyl end groups (Supplementary Figure S5D–F).

### Conservation of Hammerhead ribozyme domain in SARS-CoV-2 variants and in various species

The identification and comparison of CoV-HHRz sequences among various COVID-19 variants, including Alpha, Beta, Gamma, Delta, Lambda, and Omicron, along with an assessment of sequence conservation among different species, such as Beta-Cov (Bat CoV, Pangolin CoV, SARS-CoV, MERS-CoV) and Alpha CoV (PEDV), were conducted throughout the pandemic. The comprehensive available genome sequences of SARS-CoV-2 variants and various species were retrieved from the GISAID database. CoV-HHRz sequences were identified utilizing RNABOB and employing specific Hammerhead ribozyme search criteria, as previously outlined. Sequence alignments were performed to determine sequence identities.

**Figure 5.**
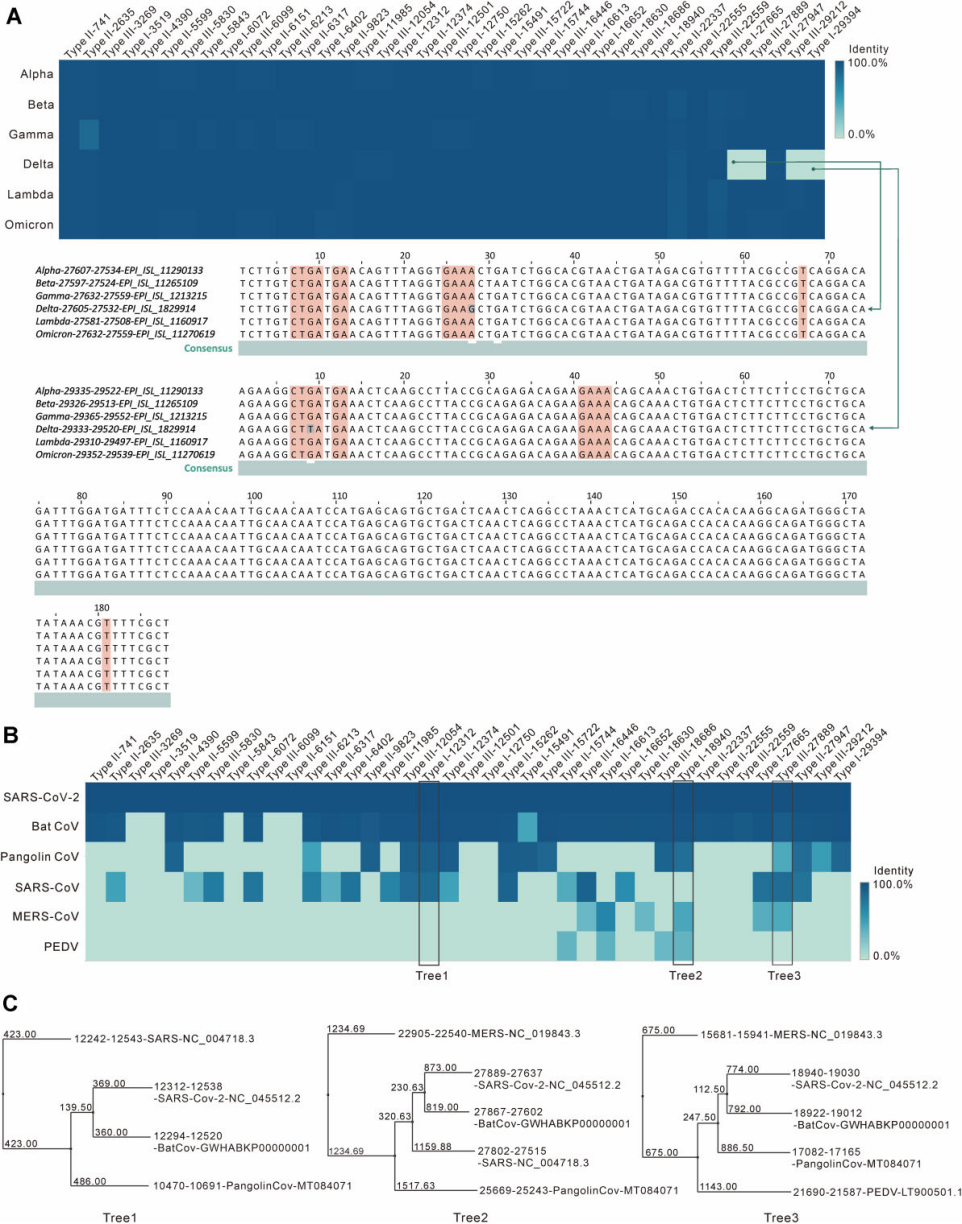
Comparison of Hammerhead-variant sequences in SARS-CoV-2 variants. (**A**) Heat Map of Hammerhead-variant Sequences in Alpha, Beta, Gamma, Delta, Lambda, and Omicron Variants. Each Hammerhead-variant sequence was compared to the Hammerhead-variant sequences in SARS-CoV-2 (NC_045512.2) using Clustal Omega. Homologies in the variant Hammerhead-variant sequences are presented as nucleotide identities, location, and hammerhead type. The identity data were used to create a heat map using ‘Tableau Public’, where each column represents a variant's Hammerhead-variant sequences and each row represents the identity of the corresponding sequence in each virus variant genome. Navy blue indicates 100% identity, while light green indicates the absence of a corresponding Hammerhead-variant sequence (defaults to 0% identity). Two sequences from the four light green boxes are displayed. These sequences were not found in the Delta genome search due to mutations in conserved nucleotides. (**B**) Heat Map of Hammerhead-variant Sequences in Six Coronaviruses: SARS-CoV-2, Bat CoV-RaTG13, Pangolin-CoV, SARS, MERS and Porcine Epidemic Diarrhoea Virus (PEDV). Each Hammerhead-variant sequence was compared to all the Hammerhead-variant sequences in SARS-CoV-2 (NC_045512.2) using Clustal Omega. Homologies in Hammerhead-variant sequences were confirmed by sequence identity and location. The identity data were used to create a heat map using Tableau Public. In this map, each column represents homologous Hammerhead-variant sequences, and each row represents the identity of the corresponding sequence in each virus genome. Navy blue represents 100% identity, while light green indicates the absence of a corresponding Hammerhead-variant sequence (defaults to 0% identity). The boxed regions highlight the sequences used to calculate the phylogenetic tree in Figure 4C. (**C**) Examples of Phylogenetic Trees of Hammerhead-variant Sequences in Coronaviruses. Calculated using Jalview 2.11.2.1.

For each SARS-CoV-2 variant, the CoV-HHRz sequences exhibit high conservation across the variants, as demonstrated in the identity heatmap presented in Figure 5A and Supplementary Table S2. Notably, four CoV-HHRz sequences in the delta variant display an exception due to a single mutation present in the conserved core hammerhead sequences, as depicted in the bottom panel of Figure 5A. The CoV-HHRz sequences between the delta and omicron variants are mostly identical, except for sequences 8, 9, 35 and 36 in omicron, which are not found in the delta variant (Supplementary Figure S6A). Supplementary Figure S6B and C show examples of the conservation of type I CoV-HHRz sequences among the variants, while Supplementary Figures S6B, D and E demonstrate conservation for type II and III.

Despite the relatively high mutation frequencies observed in the spike protein region across variants, CoV-HHRz sequences maintain a high sequence identity for type I, II, and III (Supplementary Figure S7). Although quantifying sequence variations plotted as entropy versus nucleotide position across the SARS-CoV-2 variant genomes reveals an increase in the mutation rate from 22 000 base pairs onwards (Supplementary Figure S8) (60) (Nextstrain/ncov/gisaid/global/6m), the locations of the CoV-HHRz sequences marked on an entropy vs nucleotide position bar graph suggest that most of the CoV-HHRz sequences are in regions with a low mutation rate (Supplementary Figure S8).

The identities of the 39 CoV-HHRz sequences in SARS-CoV-2 were compared with those in each of the other five species, and the results were clustered in a heatmap (Figure 5B and Supplementary Table S3). The analysis indicates that the 39 CoV-HHRz sequences in Bat CoV exhibit the highest identity with those in SARS-CoV-2, while the sequences in MERS-CoV and PEDV share the lowest identity with SARS-CoV-2 (Figure 5B). This finding aligns with the analysis of the whole genome SARS-CoV-2 sequence in comparison to other species, in the quest to trace the virus's origin (61).

Further analysis of the phylogenetic tree for three representative CoV-HHRz sequences reveals that the sequence in Bat CoV is closely grouped with that in SARS-CoV-2 (Figure 5C). However, the CoV-HHRz sequences in MERS-CoV form a distinct lineage from the others (Figure 5C). It is important to note that the lineage of sequences from different species varies depending on the different types of CoV-HHRz sequences (Figure 5C). Examples of aligned CoV-HHRz sequences for SARS-CoV-2, Bat CoV, Pangolin CoV, and SARS-CoV are shown in Supplementary Figure S9A.

The CoV-HHRz sequences from the six identified species using our search criteria were mapped onto each whole genome sequence to create a schematic representation of the CoV-HHRz sequences, displayed in Supplementary Figure S9B. The relative positions of each CoV-HHRz sequence are further analyzed and presented in Supplementary Figure S9C, illustrating the parallel relationships between some CoV-HHRz sequences in different species.

## Discussion

We have found 39 Hammerhead-variant sequences (CoV-HHRz) in the genome of SARS-CoV-2 using bioinformatics methods (Figure 1). The CoV-HHRz sequences are highly conserved within SARS-CoV-2 variants (Figure 5). These sequences in SARS-CoV-2 are closest in lineage to that of Bat Cov (Figure 5). Conventional hammerhead ribozymes are considered to function normally in the presence of added Mg^2+^ ions, although additional divalent cations can substitute for Mg^2+^ as can higher concentrations of monovalent ions (39,41,42,62). In contrast to conventional hammerhead ribozymes, the CoV-HHRz sequences found in this study are only active as ribozymes in the presence of the transition metal cations Mn^2+^, Co^2+^, Ni^2+^ or Cd^2+^ and are inactive with Mg^2+^ and monovalent ions (Figure 2). Typically, for cation dependent nucleolytic ribozymes, a correlation is observed between increasing ribozyme activity and the p*K*_a_ of each hydrated metal cation. This is consistent with an increase in the general acidity of the inner sphere water molecules of the hydrated metal ions enabling the protonation of the 5′-O leaving group resulting in an increase in ribozyme activity (38,46,63). This requirement for distinct additional divalent cations other than magnesium by the CoV-HHRz sequences supports a specific role for them in ribozyme cleavage and Mn^2+^ ions have been identified, associated with the 2′ hydroxyl of G8, in transition state analogues of the hammerhead ribozyme (40). Cleavage rates are considerably slower than those of typical hammerhead ribozymes in the presence of Mg^2+^. Otherwise, ribozyme cleavage by the CoV-HHRz-27665 ribozyme is ion and pH dependant and proceeds via a cyclic 2′,3′ phosphate intermediate, consistent with the general acid-base catalysis mechanism of a conventional hammerhead ribozyme (39,46,62). The CoV-HHRz sequences retain the core nucleotides of the ribozyme and sequence differences are limited to the neighbouring stems, which appear to be responsible for the altered cation dependence (Supplementary Figure S2 and Figure 3). Engineered enzyme strands that repair the mis-matched bases in helical stems I and III, display dramatic improvements in the observed rate constants for CoV-HHRz-27665 mutant 1 in the presence of Mn^2+^, and also recover magnesium dependence (Supplementary Figure S2 and Figure 3). The introduction of a conventional hammerhead stem loop II in CoV-HHRz-27665, mutant 2, leads to a further increase in the cleavage rate (Supplementary Figure S2 and Figure 3). The CoV-HHRz-27665 mutants 1 and 2, that rescue ribozyme activity, suggest that the role of the transition metal ions in activating the ribozyme may be associated with folding of the ribozyme into an active conformation. We also note that the base-pair at the junction of stem II is adjacent to a previously identified Mn^2+^ ion binding site (40). In cells Mn^2+^ ions are cofactors for essential enzymes, although some tissue and cell-type variation is possible, the physiological levels of Mn^2+^ have been estimated to be in the 20–60 μM range, (reviewed in (64–66)), close to the *in vitro* cleavage conditions in this study.

An additional feature of the HHRz-variant sequences is the inclusion of variable sequences (as supposed loops) between the nucleotides of helical stem II and III (Figure 1E). For example, the ribozyme activity of CoV-HHRz-5830 mutant 1 suggests that original covid sequence of stem loop II sequences is not necessarily an impediment to ribozyme activity, indeed, it is possible that sequence variations in an extended loop II, may enhance ribozyme activity through additional tertiary interactions as has been previously observed (32–34). However, inclusion of a conventional hammerhead stem loop II in CoV-HHRz in mutant 2, leads to an increase in the cleavage rate (Supplementary Figure S2 and Figure 3).

The analysis of deletion breakpoints in the expressed sub-genomic RNAs in SARS-CoV-2 transcriptome data revealed that the cleavage site of certain Hammerhead-variant ribozymes coincides with the deletion breakpoint (Figure 3, Figure 4 and Supplementary Figure S4). This alignment suggests that Hammerhead-variant ribozymes are active *in vivo*. Moreover, the deletion breakpoints in the expressed sgRNAs align with ribozyme cleavage sites, serving as footprints for this cleavage process. Notably, some deletion breakpoints were identified by labs using varying sequencing strategies, host systems, or viral isolates (Figure 3, Figure 4 and Supplementary Figure S4), providing compelling evidence of *in vivo* cleavage events.

Additionally, our study unveiled identical deletion breakpoints in different sgRNAs, indicating that subsequent events following ribozyme cleavage may vary, possibly contributing to the production of diverse sgRNAs. This finding enriches our understanding of the mechanism behind non-canonical sgRNA production, an aspect that is not yet well-understood.

In our *in vitro* biochemistry study the Hammerhead-variant ribozyme of SARS-CoV-2, retained activity when the enzyme strand and substrate strand were separated (Figure 2), and that stability in the stems through complementary base pairing significantly enhances activity (Supplementary Figures S2 and S3). Thus, it is plausible that within cells, the enzyme strand may efficiently locate favourable substrates through intermolecular or intramolecular interactions via complementary base-pairing, facilitating efficient cleavage. Similarly, small cellular molecules may enhance ribozyme cleavage *in vivo* as has been reported previously (67).

The CoV-HHRz sequences are widely distributed across the Coronaviruses (Figure 5), and are significantly enriched, compared to a random distribution (Supplementary Information). However, the relatively slow catalytic activity and dependence on transition metal ions of the CoV-HHRz suggest a limited or specialised functional role for the ribozyme. Uncontrolled ribozyme cleavage of the virus RNA would probably be deleterious to the integrity of the SARS-CoV-2 RNA genome, although a functional role for the ribozymes cannot be ruled out. Hammerhead ribozymes were first identified in the genomes of plant viruses where they performed a specific role in viral replication (21,22). Previous studies have shown that an extra nucleotide in the conventional catalytic core sequences of a viroid Hammerhead Ribozyme that causes weak ribozyme activity, is intrinsically linked to viral infectivity. Moreover, deletion of the extra nucleotide led to increased cleavage activity accompanied by a loss of viral infectivity (68). Overall, the discovery of HHRz-variant sequences across the SARS-CoV-2 genome enhances our understanding of fundamental viral biology and suggests a new function for the hammerhead ribozyme in the SARS-CoV-2 viral life cycle.

## Supplementary Material

gkae037_Supplemental_Files

## Data Availability

All supporting data are provided within the manuscript, supplementary data and Supplementary Tables S1–S4.
