# Supplementary material for: Identification of Hammerhead-variant ribozyme sequences in SARS-CoV-2
Source: Nucleic Acids Res. 2024 Jan 31;52(6):3262–77. doi: 10.1093/nar/gkae037 (PMC11014351; doi:10.1093/nar/gkae037)
Supplement: gkae037_Supplemental_Files [file gkae037_supplemental_files.zip › AASupplementary Information W_Figs.pdf]

Supplementary Information for

Identification of Hammerhead-variant ribozyme sequences in SARS-CoV-2

Getong Liu [1, 2], Hengyi Jiang [1, 2], Dongrong Chen[1, 2]* and Alastair I.H. Murchie [1,2]*

[1] Fudan University Pudong Medical Center, Pudong, Shanghai 201399, China. And Institutes of Biomedical Sciences, Shanghai Medical College, Key Laboratory of Medical Epigenetics and Metabolism, Fudan University, Shanghai 200032, China.

[2] Key Laboratory of Metabolism and Molecular Medicine, Ministry of Education, School of Basic Medical Sciences, Fudan University, Shanghai 200032, China.

Sequences Used in This Study

DNA primers for in vitro Transcription Templates	
CoV-HHRz-27665-E-F	TAATACGACTCACTATAGGTTCCTCTTGTCTGATGAACAGTTTAGGTGAAACTGAT
CoV-HHRz-27665-E-R	ATCAGTTTCACCTAAACTGTTCATCAGACAAGAGGAACCTATAGTGAGTCGTATTA
CoV-HHRz-27665-Mutant 1-E-F	TAATACGACTCACTATAGGTGCTTGTCCTCTGATGAACAGTTTCTGTGAAACGGCG
CoV-HHRz-27665-Mutant 1-E-R	CGCCGTTTCACAGAAACTGTTCATCAGAGGACAAGCACCTATAGTGAGTCGTATTA
CoV-HHRz-27665-Mutant 2-E-F	TAATACGACTCACTATAGGTGCTTGTCCTCTGATGAGTCCCAAATAGGACGAAACGGCG
CoV-HHRz-27665-Mutant 2-E-R	CGCCGTTTCGTCCTATTTGGGACTCATCAGAGGACAAGCACCTATAGTGAGTCGTATTA
CoV-HHRz-5830-E-F	TAATACGACTCACTATAGGATCAAATTTGCTGATGATTTAAACCAGTTAACTGG
CoV-HHRz-5830-E-R	TGAAGCAGGTTTCTTATAACCAGTTAACTGGTTTAAATCATCAGCAAATTTGAT
CoV-HHRz-5830-Mutant 1-E-F	TAATACGACTCACTATAGGGTATTCTGAGCTGATGATTATAACCAGTTAACTGG
CoV-HHRz-5830-Mutant 1-E-R	CAAAGTTTCTTATAACCAGTTAACTGGTTATAATCATCAGCTCAGAATAC
CoV-HHRz-5830-Mutant 2-E-F	TAATACGACTCACTATAGGGTATTCTGAGCTGATGAGTCCCAAATAGGACGAAACTTTG
CoV-HHRz-5830-Mutant 2-E-R	CAAAGTTTCGTCCTATTTGGGACTCATCAGCTCAGAATACCCTATAGTGAGTCGTATTA
CoV-HHRz-6072-E-F	TAATACGACTCACTATAGGATCAAATTTGCTGATGATTTAAACCAGTTAACTGGTTATAAGAAACCTGCTTCAAGAGAGCTTAAAGTTAC
CoV-HHRz-6072-E-R	TTTAGCTCCTTTCTTAAAAGAGGGTGTGTAGTGTTTATAATCAATAGCCACCACATCACCATTTAAGTCAGGGAAAAATGTAACTTTAAGCTCTCTTG
CoV-HHRz-6072-Mutant 1-E-F	TAATACGACTCACTATAGGTACTTCTTTGCTGATGATTAAAACCAGTTAACTGGTTATAAGAAACCTGCTTCAAGAGAGCTTAAAGTTAC
CoV-HHRz-6072-Mutant 1-E-R	CAGCCTTTCTTAAAAGAGGGTGTGTAGTGTTTATAATCAATAGCCACCACATCACCATTTAAGTCAGGGAAAAATGTAACTTTAAGCTCTCTTG

CoV-HHRz-6072-Mutant 2-E-F	TAATACGACTCACTATAGGTACTTCTTTGCTGATGAGTCCCAAATAGGACGAAAGGCTG
CoV-HHRz-6072-Mutant 2-E-R	CAGCCTTTCGTCCTATTTGGGACTCATCAGCAAAGAAGTACCTATAGTGAGTCGTATTA
CoV-HHRz-12312-E-F	TAATACGACTCACTATAGGCAGGCTAGATCTGAGGACAAGAGGGCAAAAGTTACTAGTGCTATGCAGATAATGC
CoV-HHRz-12312-E-R	TTATCCAACTTTCTAAGCATAGTGAAAAGCATTATCTGCATAGCACTAGTAAC
CoV-HHRz-12312-Mutant 1-E-F	TAATACGACTCACTATAGGCAGGTTAGGTCTGAGGACAAGAGGGCAAAAGTTACTAGTGCTATGCAGATAATGC
CoV-HHRz-12312-Mutant 1-E-R	ACAACTTTCCAAGCATAGTGAAAAGCATTATCTGCATAGCACTAGTAAC
CoV-HHRz-12312-Mutant 2-E-F	TAATACGACTCACTATAGGCAGGTTAGGTCTGAGGAGTCCCAAATAGGACGAAAGTTGT
CoV-HHRz-12312-Mutant 2-E-R	ACAACTTTCGTCCTATTTGGGACTCCTCAGACCTAACCTGCCTATAGTGAGTCGTATTA
CoV-HHRz-27889-E-F	TAATACGACTCACTATAGGTTCCTCTTGTCTGATGAACAGTTTAGGTGAAACTGATCTG
CoV-HHRz-27889-E-R	CCAGATCAGTTTCACCTAAACTGTTCATCAGACAAGAGGAA
CoV-HHRz-27889-Mutant 2-E-F	TAATACGACTCACTATAGGTCACGCCTACTGATGAGTCCCAAATAGGACGAAACGAACA
CoV-HHRz-27889-Mutant 2-E-R	TCATGTTCGTTTCGTCCTATTTGGGACTCATCAGTAGGCGTGAC
HH16-E-F	TAATACGACTCACTATAGCGATGACCTGATGAGGCCGAAAGGCCGAAACGTTCCC
HH16-E-R	GGGAACGTTTCGGCCTTTCGGCCTCATCAGGTCATCGCTATAGTGAGTCGTATTA

**Ribozyme Sequences for RtcB Ligation Fragments Preparation**	
HDVr	GGGUCGGCAUGGCAUCUCCACCUCCUCGCGGUCCGACCUGGGCUACUUCGGUAGGCUAAGGGAGAAG
HHr	GUCACGCCUACUGAUGAGUCCGUGAGGACGAAACGUGGAGACACGUC
**CoV-HHRz Fragments**	
CoV-HHRz 5' Fragment	ACAUUCUUGGUGAAAUGCAGCUACAGUUGUGAUGAUUCCUAAGAA
CoV-HHRz 3' Fragment	UAGGCGUGACAAGUUUCAUUAUGAUCUUGCAGUUCAAGUGAGAACCAAAAGAUAAUAAGCAUAAUUAAAA

	CAAGGAAUAGCAGAAAGGCUAAAAAGCACAAAUAG AAGUCAAUUAAUGAAAGUUCAAUCAUUCUGUCUUU CUUUUGAGUGUGAAGCAAAGUGUUAUAAACACUAU UGCCGCAACAAUAAGAAAAAUUGGAGAGUAAAGUU CUUGAACUUCCUCUUGUCUGAUGAACAGUUUAGGU GAAACUGAUCUGG
**DNA primers for RtcB Ligation Fragments Preparation**	
5’ Fragment-F	TAATACGACTCACTATAGGACATTCTTGGTGAAATGCA GCTAC
5’ Fragment-R	ACCTAAACTGTTCATCAGACAAGAG
3’ Fragment-F	TAATACGACTCACTATAGGTCATGTTCGTTTAGGCGTG ACAAG
3’ Fragment-R	CCAGATCAGTTTCACCTAAACTGTT
Reverse transcription primer	6-FAM CCAGATCAGTTTCACCTAAACTGTTC

**Synthesized RNA with 5’ 6-FAM Fluorescence Label**	
CoV-HHRz-27665-S	UUUACGCCGUCAGGACAAGCA
CoV-HHRz-5830-S	CUUACAAAGUCCUCAGAAUAC
CoV-HHRz-6072-S	AAACCAGUCUCUGAAGAAGUA
CoV-HHRz-12312-S	AUGGACAAUUCACCUAAUUUA
CoV-HHRz-27889-S	UCAUGUUCGUUUAGGCGUGAC
HH16-S	GGGAACGUCGUCGUCGC

The probability of the core sequence in SARS-CoV-2 genome occurring at random

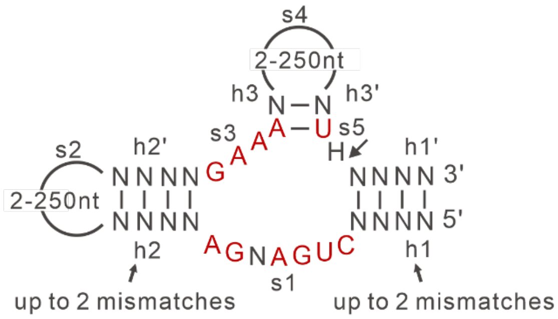

P1= P (sequence of n nucleotides appears in a genome sequence of L nucleotides)

= (L-n+1) × $\frac{B}{A}$

A (Total cases of n nucleotides sequence)
B (Matched cases of n nucleotides sequence)

There are totally (L-n+1) positions for n nucleotides sequence to appear in L nucleotides genome.

For n nucleotides sequence, there are 4 cases for each nucleotide position, therefore, total cases of n nucleotides sequence A=4^n

For n nucleotides sequence with fixed sequence, there is only one correct case, therefore B=1, the probability P1= (L-n+1) × $\frac{B}{A}$ = (L-n+1) × $\frac{1}{4^n}$

For n nucleotides sequence to have HHRz, the freedom of sequence choices are restricted by conserved regions.
Example 1, for a fixed h1, there is only 1 case of corresponding h1'.
Example 2, s2 (CUGANGA) has 4 cases.

Alterable nucleotides in HHRz including nucleotides in h1, one N in core sequence s1, nucleotides in h2, loop s2, nucleotides in h3 (except the A-U base pair), loop s4, and cleavage site s5 (H indicates 3 cases).

Therefore, B (Correct cases of n nucleotides sequence)

= $4^{h1} \times 4^{1} \times 4^{h2} \times 4^{s2} \times 4^{h3-1} \times 4^{s4} \times 3$

= $4^{4} \times 4 \times 4^{4} \times 4^{s2} \times 4 \times 4^{s4} \times 3$

= $3 \times 4^{10+s2+s4}$

P1= (L-n+1) × $\frac{B}{A}$ = (L-n+1) × $\frac{3 \times 4^{10+s2+s4}}{4^n}$

L= 30000

n= 2(h1+h2+h3)+s1+s2+s3+s4+s5

= 2(4+4+2)+7+s2+3+s4+1

= 31+s2+s4

therefore, P1=(L-n+1) × $\frac{B}{A}$

= (L-n+1) × $\frac{3 \times 4^{10+s2+s4}}{4^{31+s2+s4}}$

= (L-n+1) × $\frac{3}{4^{21}}$

When considering structured HHRz, no mismatch and extended loops.
If s2 and s4 are set as 2-20nt.

x (the minimum length of HHRz)

=2(h1+h2+h3) +s1+s3+s5+$s2_{min}$+$s4_{min}$

=2(4+4+2) + 7+3+1+2+2

=35

y (the maximum length of HHRz)

=2(h1+h2+h3) +s1+s3+s5+$s2_{max}$+$s4_{max}$

=2(4+4+2) + 7+3+1+20+20

=71

P2= P (structured HHRz in 30000nt genome)

$= \sum_{n=x}^{y} (L - n + 1)\ \frac{B}{A}$

$= \sum_{n=35}^{71} (30000 - n + 1)\ \times \frac{3}{4^{21}}$

$= \sum_{n=35}^{71} (30001 - n)\ \times \frac{3}{4^{21}}$

= [(71-35+1) × 30001 - $\frac{(71+35) \times (71-35+1)}{2}$] × $\frac{3}{4^{21}}$

=7.6 × 10^{-7}

The probability of the core sequence in SARS-CoV-2 genome occurring at three-way junctions

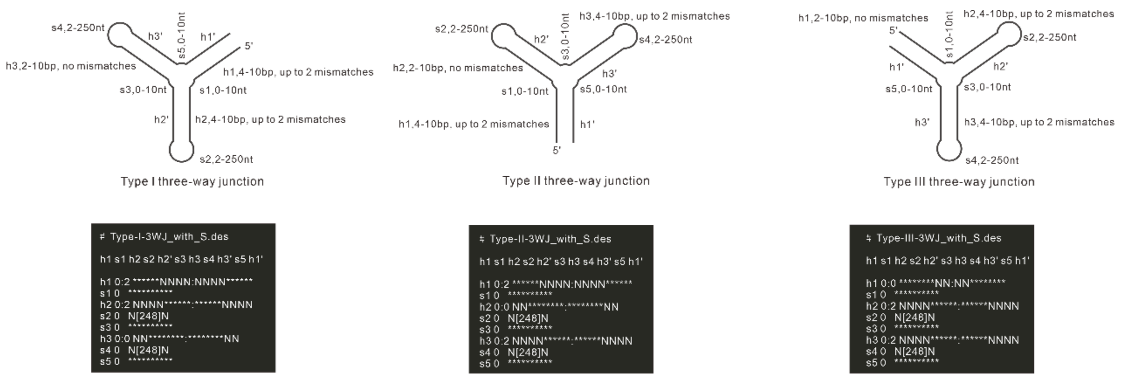

P=39/5171=7.54×10^{-3}

5171 three-way junction sequences in SARS-CoV-2 genome are displayed in supplementary document 1.

Supplementary Tables

Supplementary Table 1

Three type Hammerhead-variant sequences with location numbers in SARS-CoV-2 (NC_045512.2) genome.

Supplementary Table 2

Locations of Hammerhead-variant sequences, and the identities between Hammerhead-variant sequences in SARS-CoV-2 and its variants including Alpha, Beta, Gamma, Delta, Lambda and Omicron.

Supplementary Table 3

Locations of Hammerhead-variant sequences, and the identities between Hammerhead-variant sequences in SARS-CoV-2 and coronaviruses including Bat CoV, Pangolin CoV, SARS-CoV, MERS-CoV and PEDV.

Supplementary Table 4

Subgenomic RNA breakpoints analysis results of SARS-CoV-2 transcriptome sequencing data.

Supplementary Figures and Legends

Supplementary Figure S1

Supplementary Figure 1

```
RNABOB Descriptor
# Type I HHRz with Relax Stems and Loops.des
h1 s1 h2 s2 h2' s3 h3 s4 h3' s5 h1'

h1 0:2  **NNNN:NNNN**
s1 0    CTGANGA
h2 0:2  NNNN**:**NNNN
s2 0    N[248]N
s3 0    GAA
h3 0:0  AN**:**NT
s4 0    N[248]N
s5 0    H
```

The exemplar syntax for the hammerhead ribozyme type I.

Supplementary Figure S2

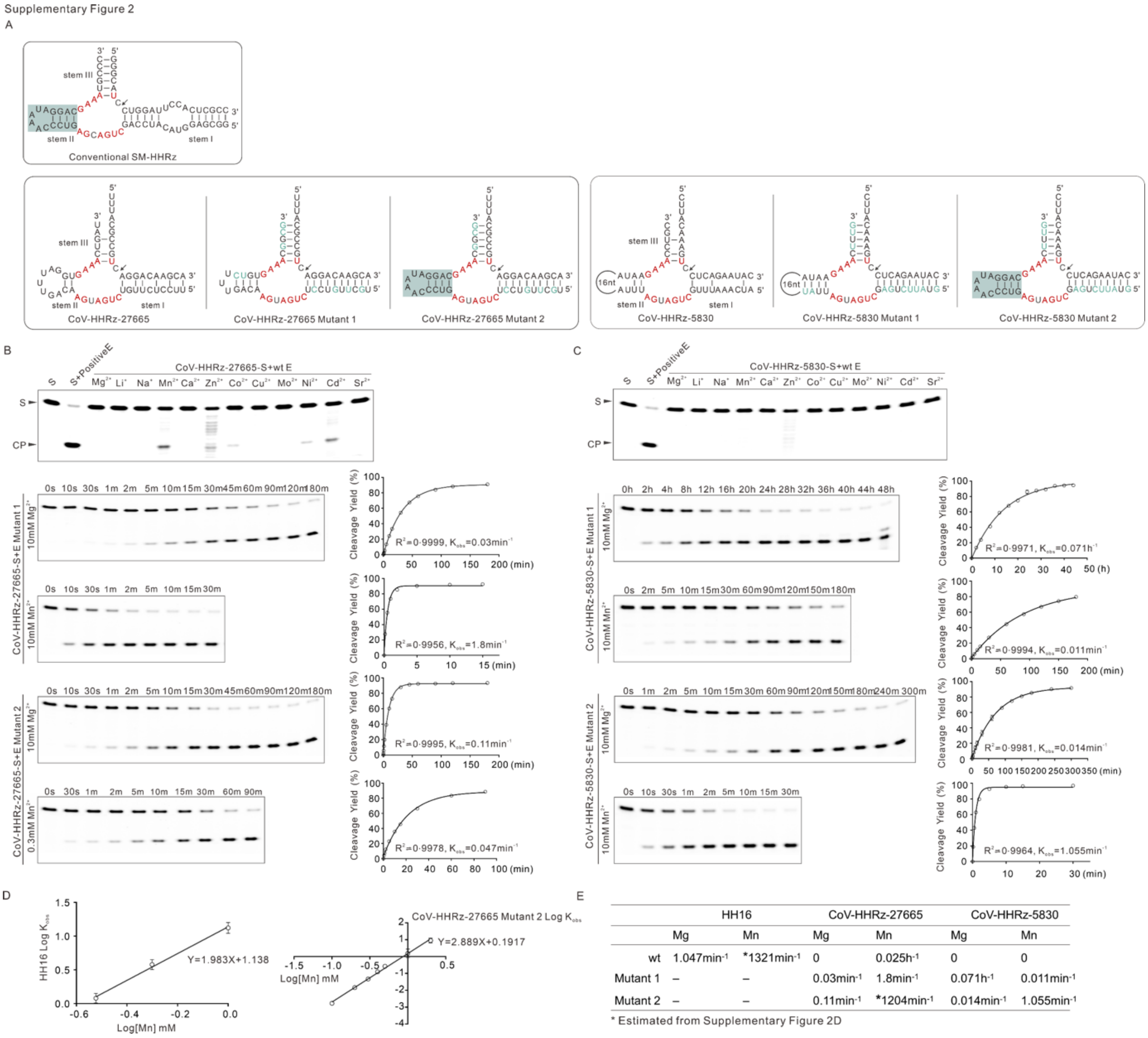

A) The constructs of conventional hammerhead ribozyme and CoV-HHRz sequences. The wild type CoV-HHRz-27665 and the inactive CoV-HHRz-5830 and mutant constructs are displayed. Mutation 1 was made by changing nucleotides in the enzyme strand to complete base-pairing in stem I, II and III. Mutation 2 further introduces stem II and loop II of the conventional hammerhead ribozyme.

B) Cleavage activity of wild type CoV-HHRz-27665 in the presence of metal ions. And the cleavage kinetics of the CoV-HHRz-27665 mutant 1 and 2. Time courses were performed at 37°C in 50 mM Tris-HCl, pH7.5, 100 mM KCl and 10 mM Mg^{2+} or 10 mM Mn^{2+} and 0.3 mM

Mn^{2+} for mutant 2. The first order rate constants (k_{obs}) of Hammerhead-variant sequence mutations were calculated by plotting the fraction of substrate cleaved (ft) versus time (t) and fitting to the equation ft = 1- exp(k_{obs}t) with GraphPad Prism 6.01. Error bars are the standard deviation of 3 independent experiments.

C) Inactive wild type CoV-HHRz-5830 in the presence of metal ions. And Cleavage kinetics of the CoV-HHRz-5830 mutant 1and 2, time courses and analysis were performed as for B except mutant 2 was incubated in 10 mM Mn^{2+}.

D) Plots of Log K_{obs} vs Log [Mn] for HH16 and CoV-HHRz 27665, the rate constants for optimal Mn^{2+} conditions (0.1, 0.2, 0.3,0.4, 0.5, 1 and 2mM Mn^{2+}) were extrapolated (34) and shown in (Supplementary Figure 2E).

E) The rate constants under optimal Mg^{2+} and Mn^{2+} conditions for the conventional hammerhead ribozyme, wild type CoV-HHRz-27665, CoV-HHRz-5830 and mutant 1 and 2.

Supplementary Figure S3

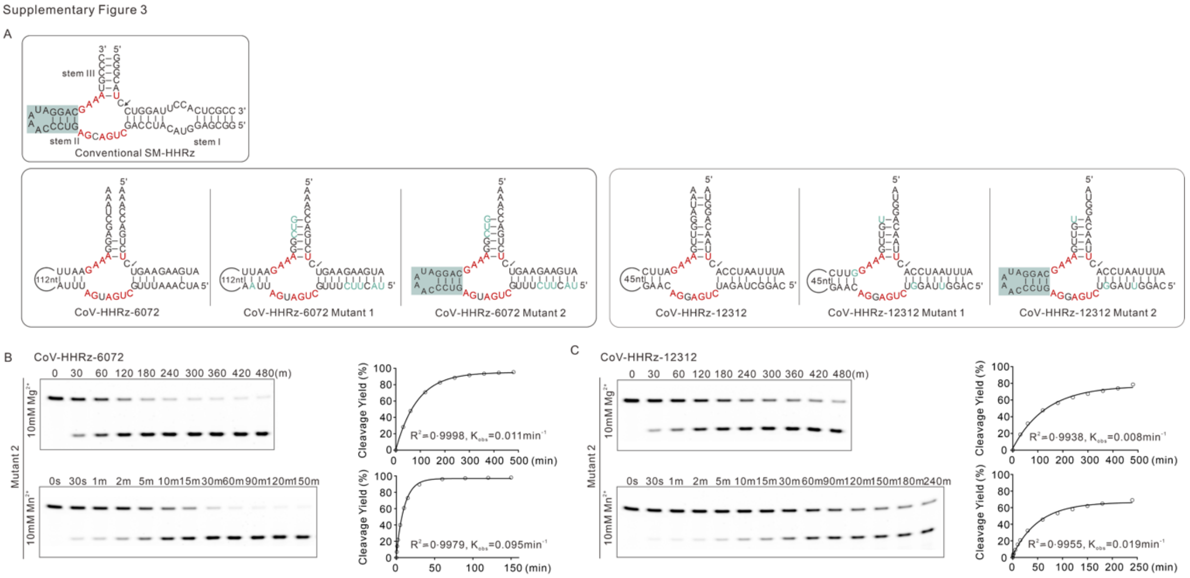

D

	CoV-HHRz-6072		CoV-HHRz-12312	
	Mg	Mn	Mg	Mn
wt	—	—	—	—
Mutant 1	—	—	—	—
Mutant 2	0.011min-1	0.095min-1	0.008min-1	0.019min-1

A) The intermolecular cleavage pattern of conventional hammerhead ribozyme and CoV-HHRz sequences. The wild type CoV-HHRz-6072 and CoV-HHRz-12312 and their mutations are displayed. Mutation 1 was obtained by changing nucleotides in enzyme strand to make stem I and III completely matched. Mutation 2 was remolded based on mutation 1 by introducing the stem II and loop II (marked by cyan box) of the conventional hammerhead ribozyme.

B) CoV-HHRz-6072 mutant 2 cleavage kinetics with magnesium or manganese ion in vitro. Time courses were performed; E + S strands were mixed and incubated at 37°C in 50mM Tris-HCl, pH7.5, 100mM KCl and 10mM Mg^{2+}, and samples removed after incubation at the given times (t). The first order rate constants (k_{obs}) of Hammerhead-variant sequence mutations were calculated by plotting the fraction of substrate cleaved (ft) versus time (t) and fitting to the equation ft = 1- exp(k_{obs}t) with GraphPad Prism 6.01. Error bars are the standard deviation of 3 independent experiments.

C) CoV-HHRz-12312 mutant 2 cleavage kinetics with magnesium or manganese ion in vitro.

D) Rate constants of CoV-HHRz-6072 and CoV-HHRz-12312 wild type, mutant 1 and mutant 2.

Supplementary Figure S4

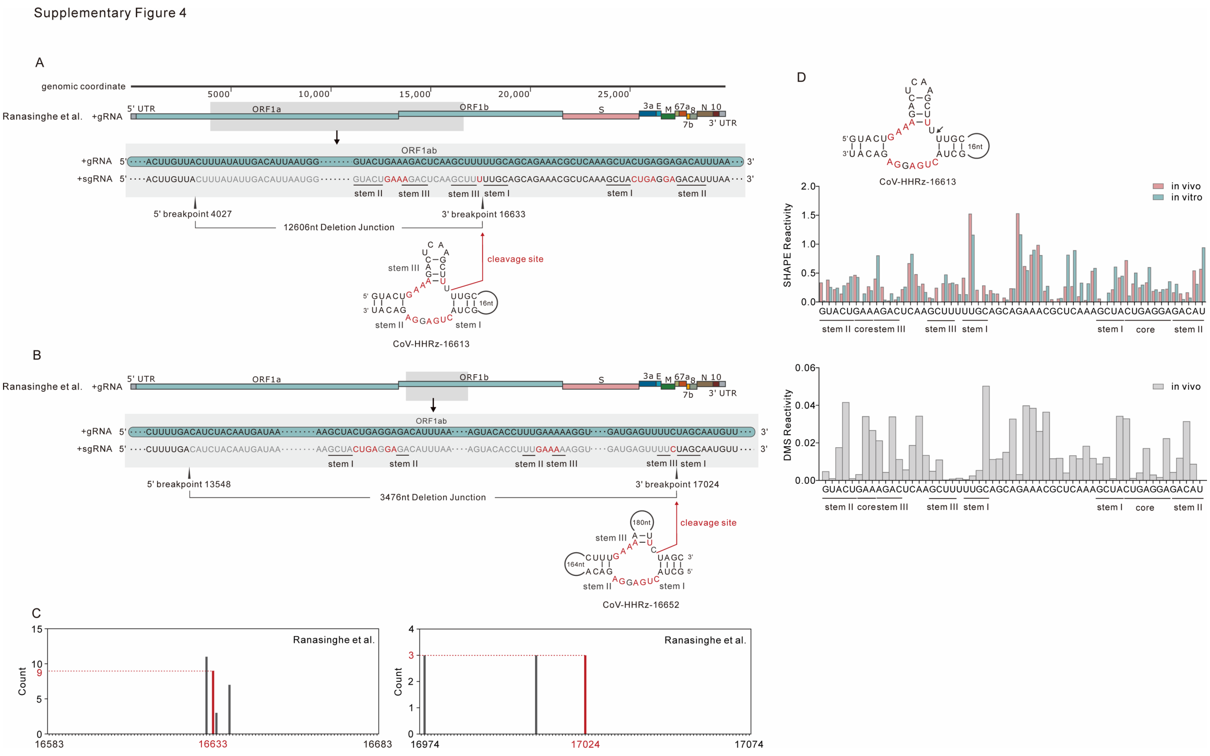

A) The relationship between the location of CoV-HHRz-16613 and the breakpoints of subgenomic RNA identified by Ranasinghe et al. The regions involved are highlighted with grey shadows. The 5' and 3' breakpoints of subgenomic RNA are marked by black triangles, the deleted junction fragment is shown. The stems of the CoV-HHRz sequence are marked. The conserved bases within HHRz are marked in red. The red arrow represents the cleavage site. The deleted nucleotides are represented in grey.

B) The relationship between the location of CoV-HHRz-16652 and the breakpoints of subgenomic RNA identified by Ranasinghe et al. The breakpoints, deleted junctions, HHRz stems, conserved bases and cleavage site are highlighted as described above.

C) The histograms display the count of detected breakpoints at 16633 and 17024 (in red) compared to the neighbouring the ±50 nucleotides. The nucleotide position for each breakpoint is represented by a red bar, and the counts denoted in red and by red dotted reference lines.

D) Chemical probing data of CoV-HHRz-16613. The folding pattern of CoV-HHRz-16613 RNA and the SHAPE reactivity measured by Manfredonia et al. The *in vivo* and *in vitro* SHAPE reactivity are displayed as pink and pea green respectively. *In vivo* DMS reactivity of

CoV-HHRz-16613 RNA as measured by Lan et al. is displayed in grey. The stems and core sequences of CoV-HHRz-16613 are shown.

Supplementary Figure S5

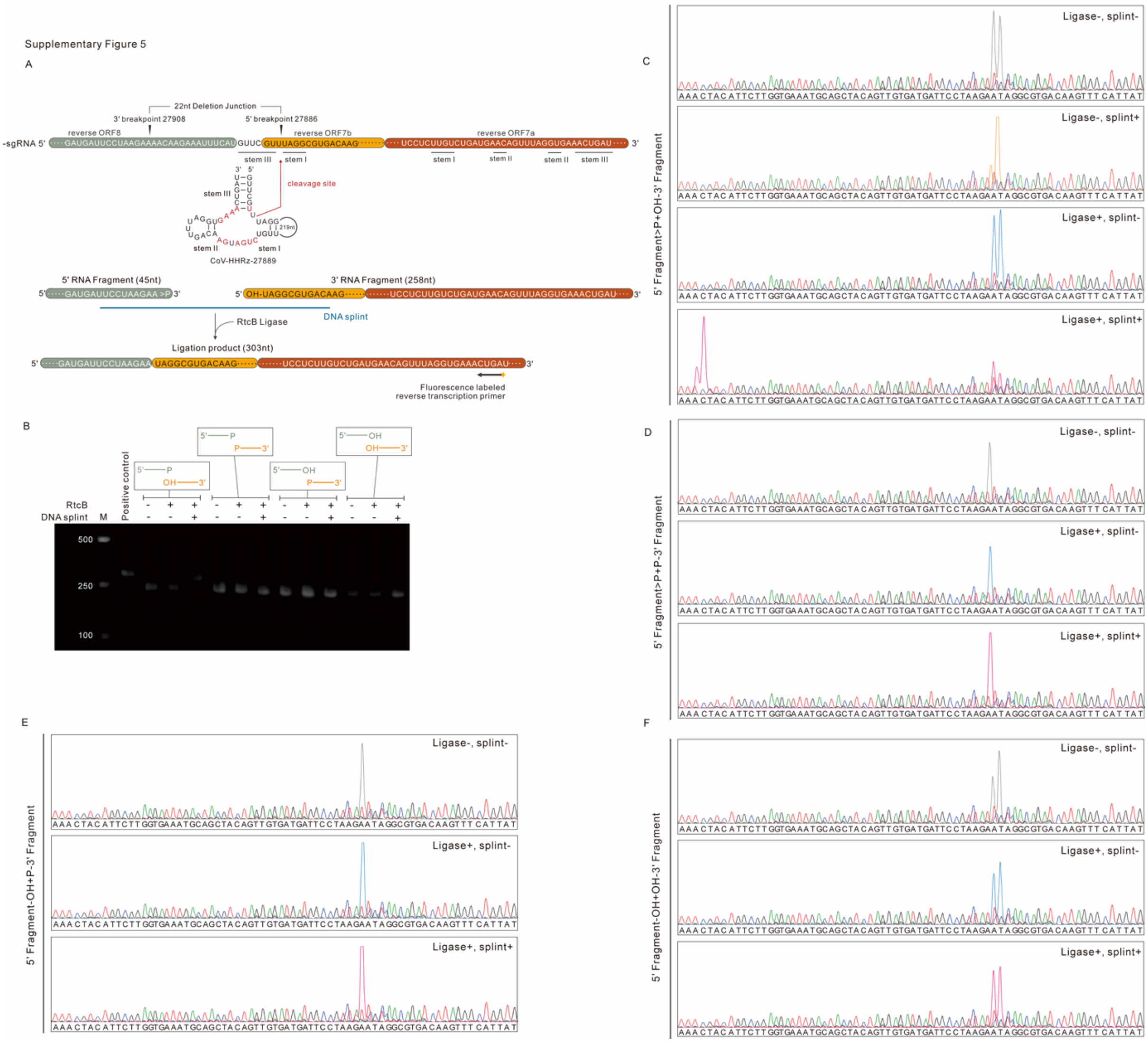

A) Schematic for the deletion and ligation of sequences flanking the 5’ cleavage site identified by Kim and colleagues (15). **B)** 1% agarose gel electrophoresis of the reverse transcribed cDNA ligation products. The ligations are grouped as 5’ and 3’ fragments, such that: 5’ fragment with 2’,3’-cyclic phosphate end is ligated with 3’ fragment with 5’ hydroxyl end; 2’,3’-cyclic phosphate with 5’ phosphate; 3’ hydroxyl with 5’ phosphate; 3’ hydroxyl with 5’ hydroxyl. The positive marker is the cDNA reverse transcribed by using purified ligated RNA as template. **C)** Capillary sequencing of 5’ fragment with 2’,3’-cyclic phosphate end + 3’ fragment with hydroxy end group (as in B). **D)** Sequencing of 5’ fragment with 2’,3’-cyclic phosphate end with 3’ fragment with 5’ phosphate (as in B). **E)** Sequencing of 5’ fragment with 3’ hydroxyl end with 3’ fragment with 5’ phosphate (as in B). **F)** Sequencing of 5’ fragment with 3’ hydroxyl end with 3’ fragment with 5’ hydroxyl end group (as in B).

Supplementary Figure S6

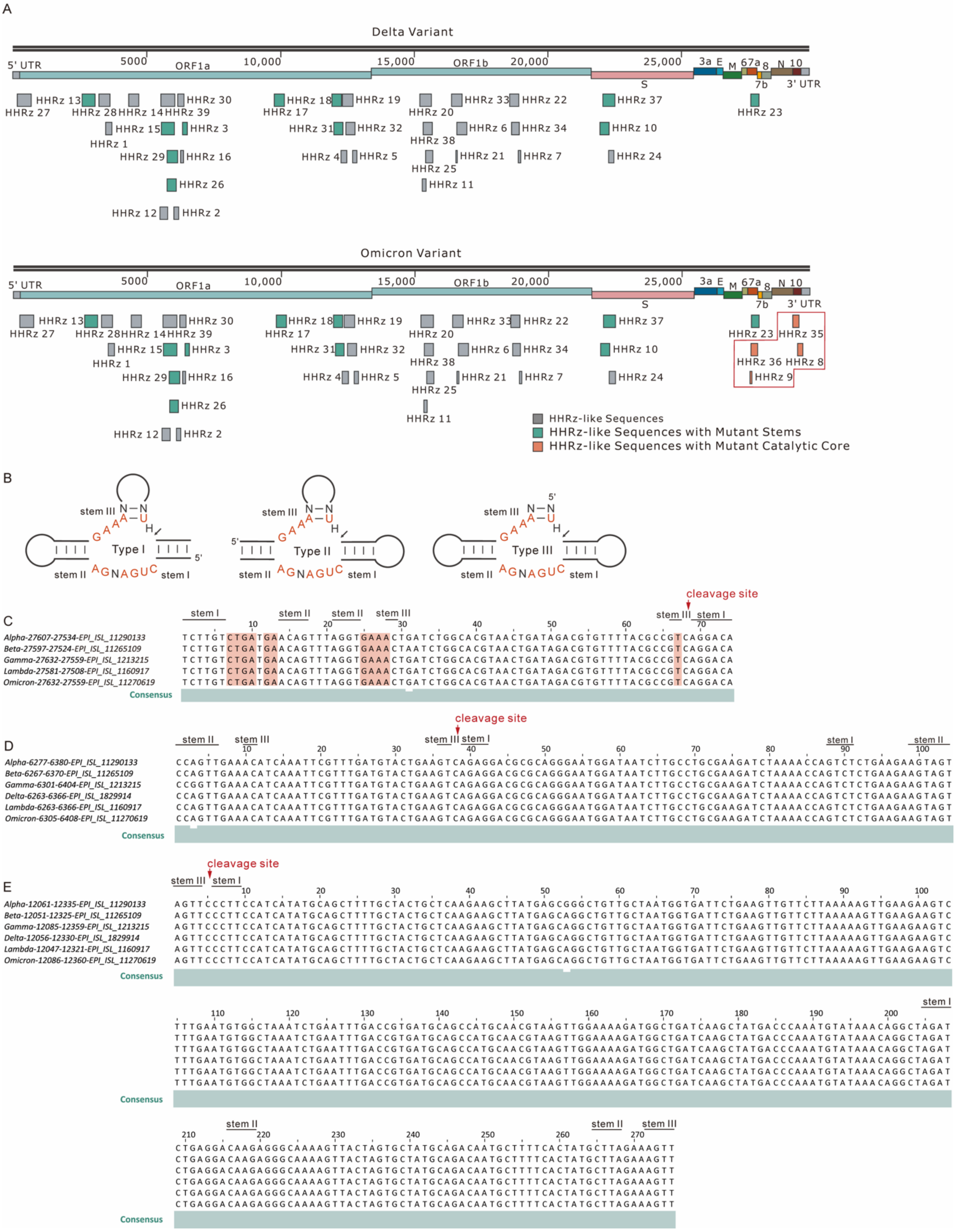

A) Schematic of Hammerhead-variant sequences in the Delta variant and Omicron variants. The genome locations and annotations are displayed. The locations of the Hammerhead-variant sequences were show by coloured boxes. Grey boxes represent identical sequences and green

boxes represent sequences with mutations in non-conserved stems/loops. **B)** Potential folding pattern of Type I/III/III hammerhead ribozyme sequences. **C)** The alignment of exemplar type I Hammerhead-variant sequences in SARS-CoV-2 variants genome. Cyan panel represents the identity among the sequences. Possible stems are labeled. Possible cleavage sites are marked by red arrow. **D)** The alignment of exemplar type II Hammerhead-variant sequences in SARS-CoV-2 variants genome. The identity, CoV-HHRz stems and cleavage site are shown as described above. **E)** The alignment of exemplar type III Hammerhead-variant sequences in SARS-CoV-2 variants genome. The identity, CoV-HHRz stems and cleavage site are shown as described above.

Supplementary Figure S7

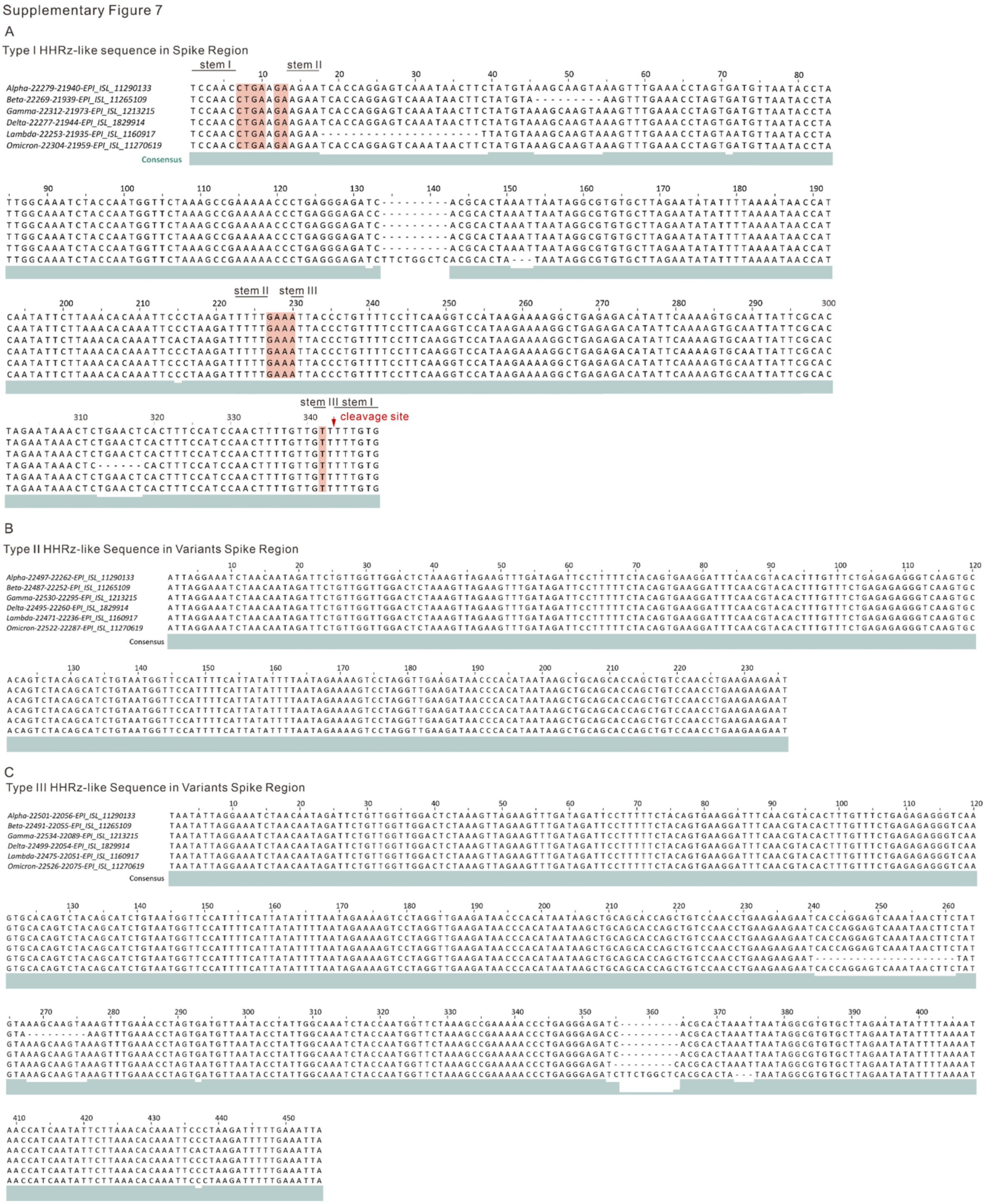

A) The alignment of exemplar type I Hammerhead-variant sequences in SARS-CoV-2 variants genome Spike ORF region. Cyan panel represents the identity among the sequences. Possible stems are labeled. Possible cleavage sites are marked by red arrow.

B) The alignment of exemplar type II Hammerhead-variant sequences in SARS-CoV-2 variants

genome Spike ORF region. **C)** The alignment of exemplar type III Hammerhead-variant sequences in SARS-CoV-2 variants genome Spike ORF region

Supplementary Figure S8

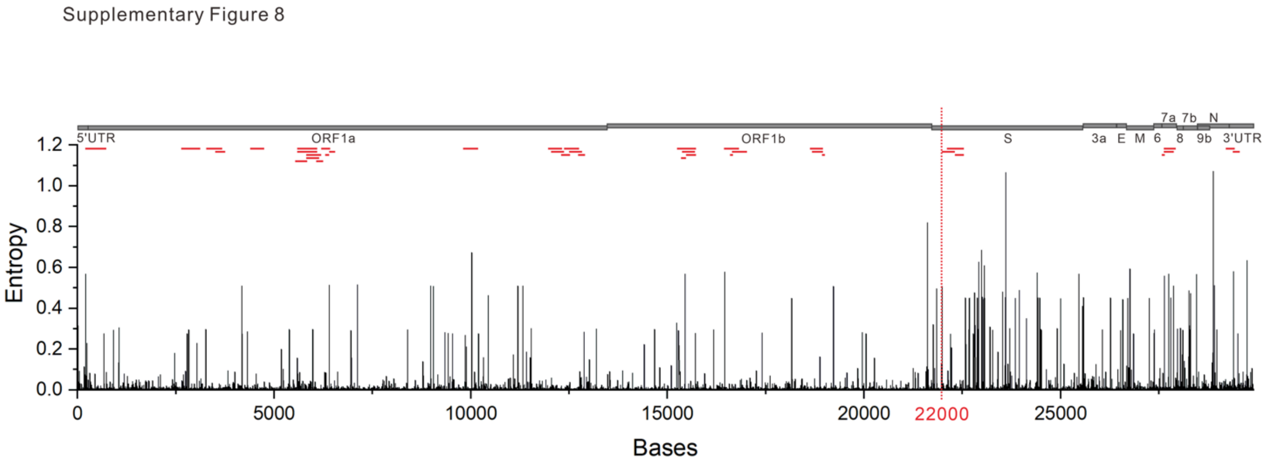

Comparison between the location of CoV-HHRz-like sequences and the variable SARS-CoV-2 genome nucleotides. The red transverse lines represent regions covered by CoV-HHRz-variant sequences. The bar graph that represents the entropy of nucleotide diversity along the SARS-CoV-2 genome, obtained from the Nextstrain website (Nextstrain / ncov / gisaid / global / 6m). The red dotted line indicates the location 22000 on SARS-CoV-2 genome.

Supplementary Figure S9

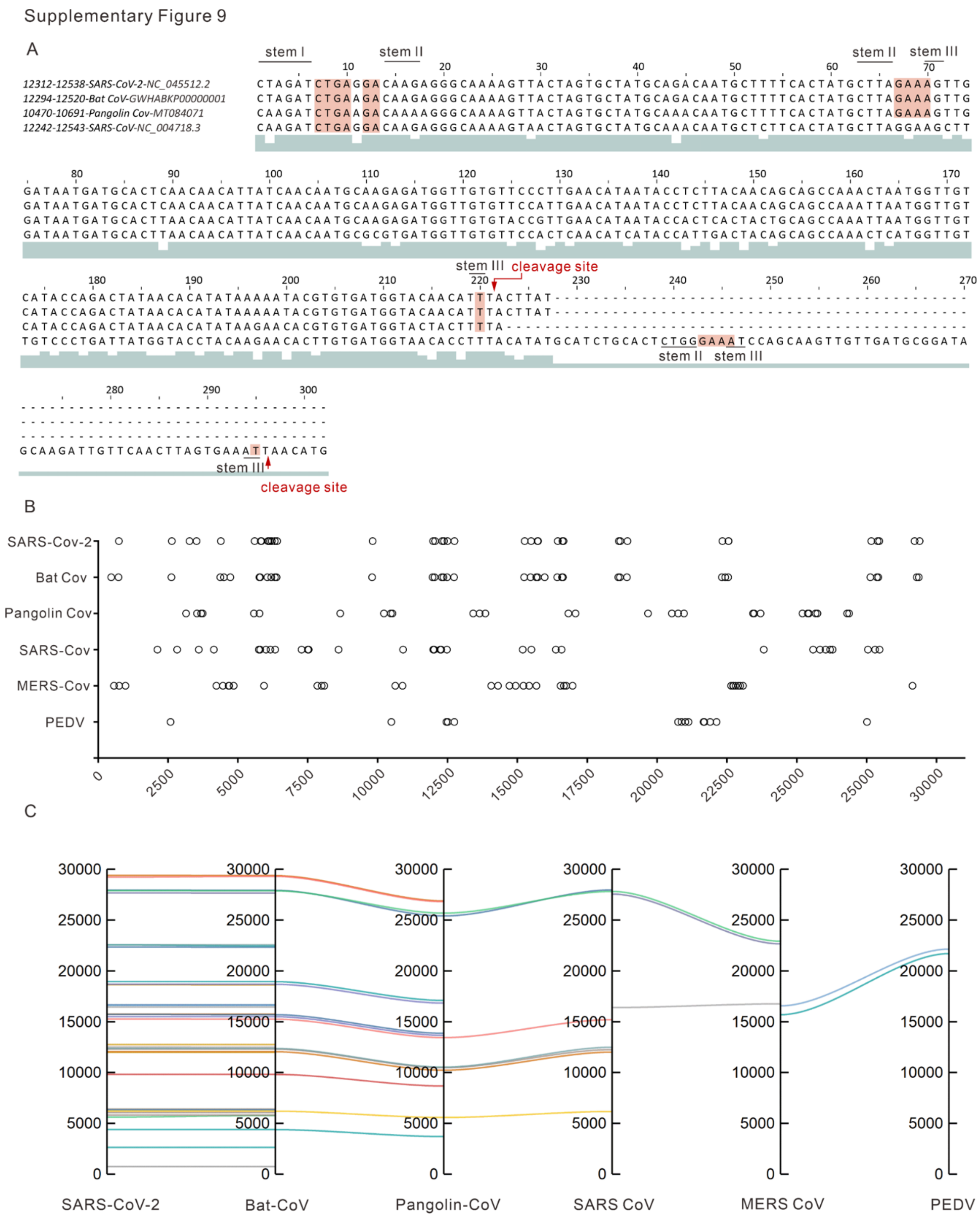

A) Alignment of the CoV-HHRz-12312 in SARS-CoV-2 genome and its parallel sequences in Bat CoV-RaTG13, Pangolin CoV and SARS-CoV. The accession numbers and location numbers are shown. Conserved nucleotides are highlighted by pink boxes. The red arrowhead indicates the cleavage site. Nucleotides that are included in stems I-III in the RNABOB search

output are marked by horizontal lines. **B)** Distribution of Hammerhead-variant sequences in SARS-CoV-2, Bat CoV-RaTG13, Pangolin CoV, SARS-CoV, MERS-CoV and Porcine epidemic diarrhoea virus (PEDV). Each hollow circle represents location of the corresponding Hammerhead-variant sequences start site in each viral genome. **C)** Parallel plot of clustering of Hammerhead-variant sequences in SARS-CoV-2 and other coronavirus. Lateral lines show Hammerhead-variant sequences sharing both similar location and sequence identity. The Hammerhead-variant sequences are present in each of the six species with their relative positions slightly skewed. Some of the Hammerhead-variant sequences share a certain level of identity with their counterparts in different species while others are present independently.
